# Continuous-flow hydration–condensation reaction: Synthesis of α,β-unsaturated ketones from alkynes and aldehydes by using a heterogeneous solid acid catalyst

**DOI:** 10.3762/bjoc.7.198

**Published:** 2011-12-15

**Authors:** Magnus Rueping, Teerawut Bootwicha, Hannah Baars, Erli Sugiono

**Affiliations:** 1 Institute of Organic Chemistry, RWTH Aachen University, Landoltweg 1, D-52074 Aachen, Germany

**Keywords:** chalcones, flow reactor, green chemistry, heterogeneous catalysis, microwave

## Abstract

A simple, practical and efficient continuous-flow hydration–condensation protocol was developed for the synthesis of α,β-unsaturated ketones starting from alkynes and aldehydes by employing a heterogeneous catalyst in a flow microwave. The procedure presents a straightforward and convenient access to valuable differently substituted chalcones and can be applied on multigram scale.

## Introduction

In recent years, the development of continuous-flow technologies has expanded considerably and has had a significant impact on modern organic synthetic chemistry. Continuous-flow processes offer advantages, such as operational simplicity, energy savings, reduced reagent consumption, and improved mixing quality as well as precise control of the reaction parameters, including pressure, temperature, residence time and heat transfer. The improved operational safety over classical batch reactions reduces the problems of working with hazardous chemicals [1–22]. Furthermore, continuous-flow technologies allow chemical processes to be easily and rapidly scaled up, either by changing the volume of the single reactor, by performing the reaction for an extended reaction time, or by running the reaction in multiple reactors in parallel. Moreover, the products may be collected continuously and formation of byproducts may be reduced by the immediate separation of the products from the reaction mixtures [23–34]. More recently, it has been shown that even asymmetric reactions can be conducted in a continuous-flow fashion [35–40]. Recently, the combination of flow processes and microwave technology has become an interesting endeavour in both academia and in industry. The combination of continuous-flow technology and microwave heating offers advantages such as a cleaner reaction profile, reduction of reaction times, higher yields and better selectivity [41–54].

## Results and Discussion

α,β-Unsaturated ketones are a common motif found in the principal core of a large number of important biologically active compounds. They show pharmacological properties such as antimalarial, antitumor, antiviral, and anti-inflammatory activities [55–60]. They are also well known to be key intermediates in the synthesis of flavones, flavonoids, isoflavonoids and other heterocyclic compounds [61–65]. Consequently, the development of an efficient synthesis to obtain these valuable compounds attracted our interest.

Thus, we decided to develop an efficient continuous-flow synthesis of α,β-unsaturated ketones starting from alkynes and aldehydes by employing a heterogeneous solid acid catalyst [66–84].

**Scheme 1:**
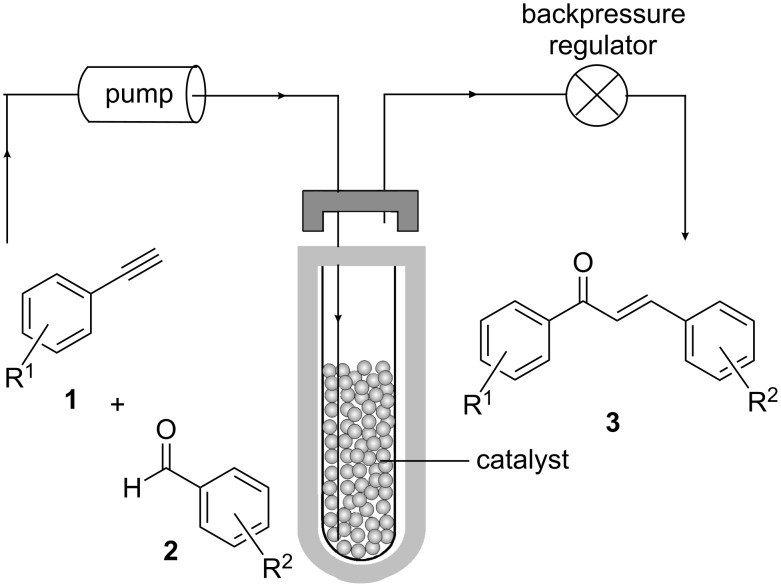
The schematic arrangement of the continuous-flow system.

The continuous-flow apparatus for the experiment was set up according to Scheme 1. A 10 mL reaction vessel was charged with the heterogeneous solid acid catalyst (10 g) and inserted into the reactor. By means of a peristaltic pump the reagents were continuously pumped through the reaction vessel under microwave irradiation (50 W). The product solution was collected from the outlet tube, which was connected to a 250 psi backpressure regulator of the commercial flow microwave system.

Our initial reaction development was focussed on finding the optimal conditions for the continuous-flow reaction of phenylacetylene (**1a**) with benzaldehyde (**2a**) applying the ion-exchange resin amberlyst-15 [85] as heterogeneous solid acid catalyst. The effects of the substrate concentration, the reaction temperature and the flow rate are summarized in Table 1. Performing the reaction at 80 °C afforded the α,β-unsaturated ketone in 70% yield (Table 1, entry 5). Running the reaction at higher temperature (90 °C) showed a noticeable impact on the conversion and the product was isolated in 84% (Table 1, entry 2). However, a further temperature increase did not result in improved yield (Table 1, entry 1). Next, we set out to probe the influence of the flow rate on the chemical yield. Performing the reaction at 90 °C and 0.5 mL min^−1^ gave the product in 84% yield (Table 1, entry 2). A significant drop in chemical yield was observed when the flow rate increased from 0.5 mL min^−1^ to 1.0 mL min^−1^ (Table 1, entry 2 versus entry 8), indicating that the flow rate should be 0.5 mL min^−1^. Further reaction optimization was accomplished by varying the substrate concentration. While the chemical yields remained constant during an increase from 0.2 M to 0.3 M (Table 1, entries 2 and 7), performing the reaction at lower concentration (0.1 M) afforded the product in lower yield (Table 1, entry 6). A slightly better conversion was obtained when the reaction was performed in dry solvent (Table 1, entry 3). However, the best result was achieved when the reaction was conducted under solvent-free conditions (Table 1, entry 4).

**Table 1:** Optimization of hydration–condensation reactions.^a^

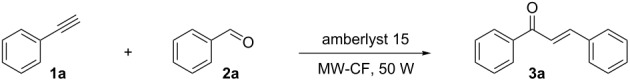					

Entry	Flow rate [mL min^−1^]	Conc. **1a** [M]	Heat Source	Temp. [°C]	Yield [%]^b^

1	0.5	0.2	microwave	100	84
2	0.5	0.2	microwave	90	84
3	0.5	0.2	microwave	90	87^c^
4	0.5	–	microwave	90	91^d^
5	0.5	0.2	microwave	80	70
6	0.5	0.1	microwave	90	62
7	0.5	0.3	microwave	90	84
8	1.0	0.2	microwave	90	30
9^e^	–	0.1	–	35	n.r.
10^f^	–	0.2	oil bath	80	17
11^g^	–	0.2	oil bath	80	47

^a^Reaction conditions: Phenylacetylene (**1a**) (1.0 equiv), 0.2 M in 1,2-dichloroethane (DCE) and benzaldehyde (**2a**) (4.0 equiv), 50 W, 30 min. ^b^Isolated yield after column chromatography. ^c^Performed with dry 1,2-dichloroethane (DCE). ^d^Performed under neat (solvent-free) conditions. ^e^Performed in DCM under batch conditions for 24 h. ^f^Performed in DCE under batch conditions for 1 h. ^g^Performed in DCE under batch conditions for 3 h.

To probe the influence of microwave heating on this transformation, we examined the same transformation under batch conditions and without microwave irradiation. As shown in Table 1, under classical batch conditions no product formation was observed when the reaction was performed at 35 °C (Table 1, entry 9). By increasing the reaction temperature to 80 °C using an oil bath, 17% of the product was isolated after 1 hour reaction time (Table 1, entry 10). Higher isolated yield (47%) was obtained with a longer reaction time (3 h) (Table 1, entry 11). Identical transformation under microwave conditions gave 70% isolated yield after 30 min reaction time (Table 1, entry 5).

Having established the optimal reaction conditions, we set out to investigate the scope and applicability of the procedure by employing various alkynes **1** and a range of substituted aromatic aldehydes **2** [86]. All the reactions were performed neat, except where the aldehydes were solid. In those cases the reactions were performed in dry DCE. The results are summarized in Table 2.

**Table 2:** Flow hydration–condensation of alkynes **1** and aldehydes **2**.^a^

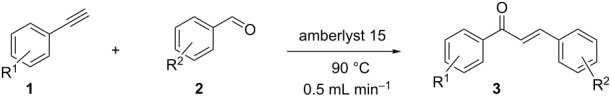				

Entry	Alkyne **1**	Aldehyde **2**	Product **3**	Yield [%]^b^

1	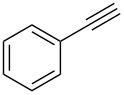 **1a**	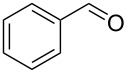 **2a**	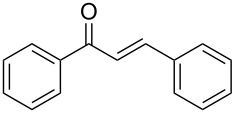 **3a**	87^c^ 91
2^c^	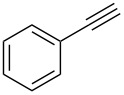 **1a**	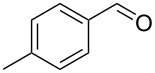 **2b**	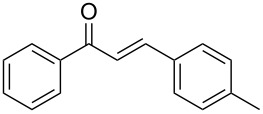 **3b**	96
3^c^	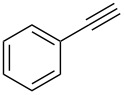 **1a**	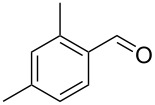 **2c**	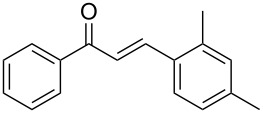 **3c**	98
4	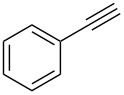 **1a**	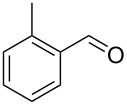 **2d**	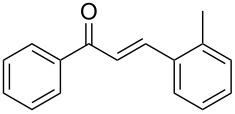 **3d**	87
5	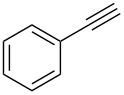 **1a**	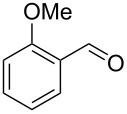 **2e**	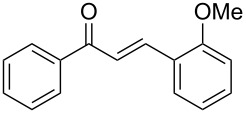 **3e**	85
6^c^	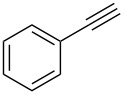 **1a**	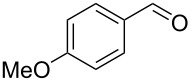 **2f**	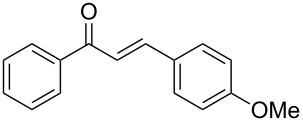 **3f**	89
7	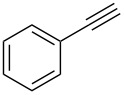 **1a**	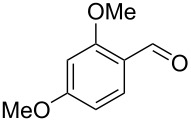 **2g**	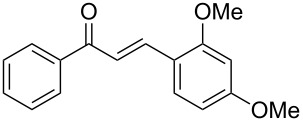 **3g**	88
8	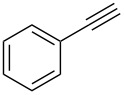 **1a**	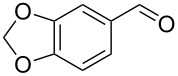 **2h**	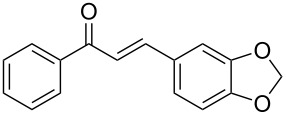 **3h**	95
9	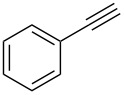 **1a**	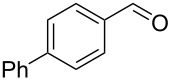 **2i**	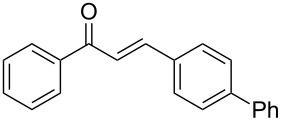 **3i**	86
10^c^	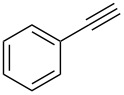 **1a**	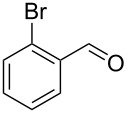 **2j**	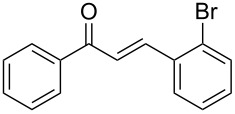 **3j**	82
11^c^	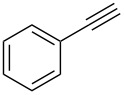 **1a**	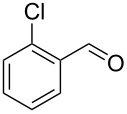 **2k**	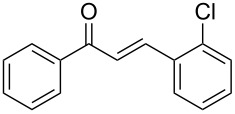 **3k**	77
12	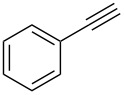 **1a**	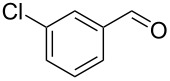 **2l**	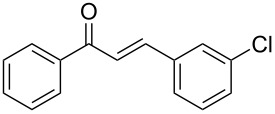 **3l**	68
13	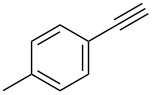 **1b**	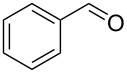 **2a**	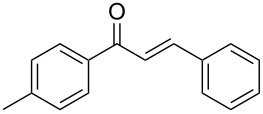 **3m**	98
14	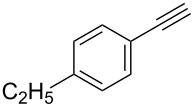 **1c**	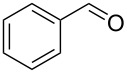 **2a**	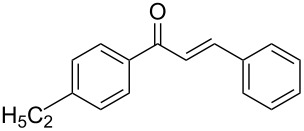 **3n**	97
15	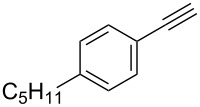 **1d**	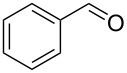 **2a**	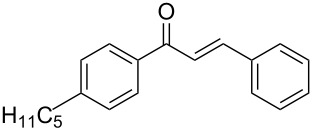 **3o**	83
16	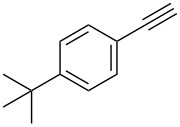 **1e**	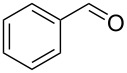 **2a**	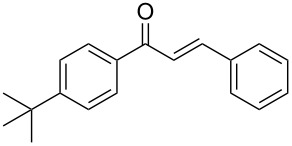 **3p**	91
17	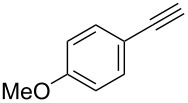 **1f**	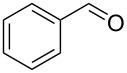 **2a**	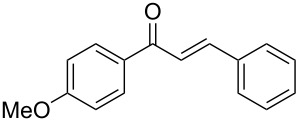 **3q**	73
18	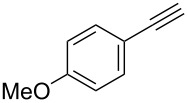 **1f**	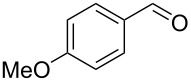 **2f**	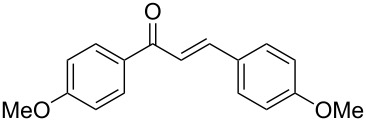 **3r**	79
19	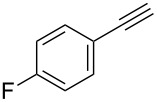 **1g**	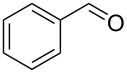 **2a**	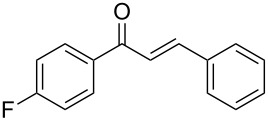 **3s**	70
20	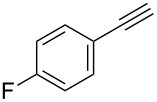 **1g**	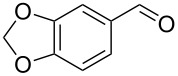 **2h**	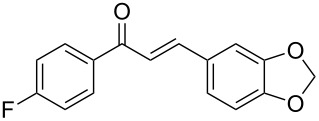 **3t**	77
21	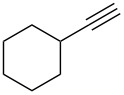 **1h**	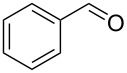 **2a**	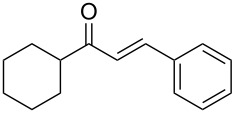 **3u**	74

^a^Reaction conditions: alkynes **1** (1 equiv), aldehydes **2** (4 equiv), solvent-free conditions, 50 W. ^b^Isolated yield after column chromatography. ^c^Performed in dry 1,2-dichloroethane (0.2 M).

Generally, the reaction mixture of alkyne **1** and aldehyde **2** was constantly pumped into the flow cell, filled with the solid acid catalyst and solvent, at the flow rate of 0.5 mL min^−1^ under microwave irradiation. This was followed by a washing procedure with 100 mL of solvent and then the next substrate was introduced. Importantly, the same catalyst was maintained throughout the reactions.

In general, various aldehydes bearing electron-withdrawing or -donating groups, as well as different substitution patterns were suitable substrates in the reactions. The corresponding α,β-unsaturated ketones were obtained in good to excellent yields (Table 2, entries 1–12). Applying this procedure no formation of propargylic alcohols was observed and in general no amount of methyl ketone was detected.

Employing the above optimized reaction conditions, further experiments were conducted with a range of substituted alkynes **1** and aldehydes **2** (Table 2, entries 13–21). Again, both electron-rich and electron-poor substrates were well tolerated and the corresponding products were isolated in good yields.

**Scheme 2:**
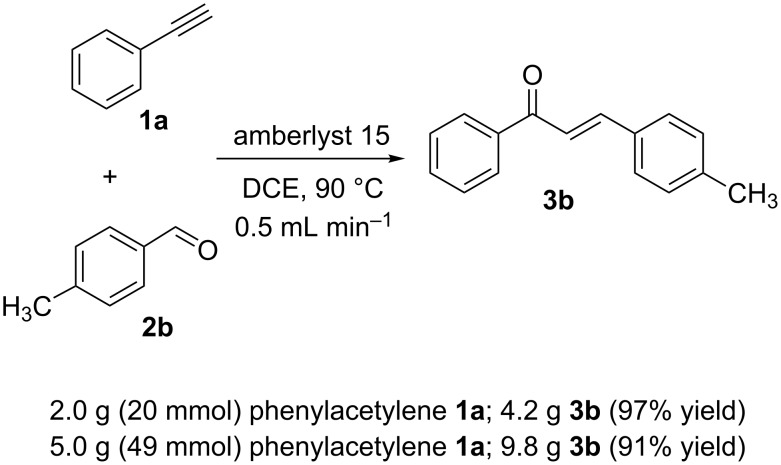
Preparation of chalcone **3b** on larger scale.

Once the optimal reaction conditions were successfully established on a small scale, we evaluated the potential of this protocol by performing the reactions on a 20 and 49 mmol scale. The reactions of phenylacetylene (**1a**) with *p*-methyl substituted benzaldehyde **2b** proceeded smoothly providing the corresponding product in excellent isolated yields (Scheme 2).

## Conclusion

In conclusion, we have developed a general protocol to access a series of valuable differently substituted chalcones. Starting from commercially available alkynes and aldehydes, a continuous-flow hydration–condensation protocol leads to the desired products in good to excellent yields. The reactions were sequentially introduced into the flow cell and performed several times without the heterogeneous catalyst needing to be changed, demonstrating the high robustness of this catalytic system. Additionally, this new method was readily applied for the preparation of chalcones in multigram quantities. The technology presented is advantageous over classical non-microwave batch reactions in particular with regard to the continuous harvesting of the product, the fast optimization of the reaction parameters, the simple operation and reliability, and the restriction of byproduct formation, especially the formation of methyl ketones and propargylic alcohols.

## Experimental

General procedure for hydration–condensation reaction of phenylacetylene (**1a**) and benzaldehyde (**2a**). A solution of phenylacetylene (**1a**) (0.4 mmol) and benzaldehyde (**2a**) (1.6 mmol) was pumped through the flow cell filled with amberlyst-15 resin (16–50 mesh) (10.0 g) and dry 1,2-dichloroethane at a flow rate of 0.5 mL min^−1^. During this period the reaction vessel in a microwave cavity was irradiated at 90 °C (50 W). Following the reaction, 100 mL solvent was pumped through the flow cell at the same flow rate in order to wash the system, and the combined solutions were evaporated in vacuo. The residue was purified by column chromatography (*n*-hexane/DCM [2:1 to 1:1]).

## Supporting Information

File 1 Experimental procedures and characterization of compounds.
