# Supplementary material for: Continuous-flow hydration–condensation reaction: Synthesis of α,β-unsaturated ketones from alkynes and aldehydes by using a heterogeneous solid acid catalyst
Source: Beilstein J Org Chem. 2011 Dec 15;7:1680–7. doi: 10.3762/bjoc.7.198 (PMC3252873; doi:10.3762/bjoc.7.198)
Supplement: File 1 — Experimental procedures and characterization of compounds. [file Beilstein_J_Org_Chem-07-1680-s001.pdf]

Supporting Information

for

Continuous-flow hydration–condensation reaction: Synthesis of α,β-unsaturated ketones from alkynes and aldehydes by using a heterogeneous solid acid catalyst

Magnus Rueping*, Teerawut Bootwicha, Hannah Baars and Erli Sugiono

Address: Institute of organic chemistry, RWTH Aachen University, Landoltweg 1, D-52074 Aachen, Germany.

Email: Magnus Rueping* - magnus.rueping@rwth-aachen.de

* Corresponding author

Experimental procedures and characterization of compounds.

General: Unless otherwise noted, all commercially available compounds were used as received. Solvents for chromatography were technical grade and distilled prior to use. Dry 1,2-dichloroethane (DCE) used in reactions was obtained by distilling over calcium hydride and was stored over activated molecular sieves (4 Å). Analytical thin-layer chromatography (TLC) was performed on Merck silica gel 60 aluminium plates with F-254 indicator, visualised by UV irradiation. Column chromatography was performed on Macherey-Nagel silica gel (particle size 0.040–0.063 mm). ^{1}H NMR and ^{13}C NMR were recorded on a Mercury 300 or Inova 400 spectrometer in $CDCl_3$ with the residual proton signal of the deuterated solvents as the internal reference (δ_H = 7.26 ppm and δ_C = 77 ppm for $CDCl_3$). Data are

reported in the following order: chemical shift (δ) in ppm; multiplicities are indicated s (singlet), d (doublet), t (triplet), q (quartet), m (multiplet), dd (doublet of doublet), tt (triplet of triplet), dt (doublet of triplet); coupling constants (*J*) are in Hertz (Hz). (MS-EI, 70 eV) were conducted on GC-MS Shimadzu QP2010 (column: Equity®-5, length × I.D. 30 m × 0.25 mm, d_f 0.25 μm, lot # 28089-U, Supelco). All experiments were performed with Voyager flow microwave reactor (CEM) equipped with peristaltic pump and backpressure regulator.

General procedure for in-flow synthesis of chalcones.

Preparation of (*E*)-1,3-diphenyl-2-propen-1-one (3a).

A solution of phenylacetylene (0.4 mmol) and benzaldehyde (1.6 mmol) was pumped through the flow cell filled with amberlyst-15 resin (10.0 g) and 1,2-dichloroethane, at a flow rate of 0.5 mL min^{-1}. During this period the flow reactor was irradiated in a microwave cavity at 90 °C (50 W). In order to wash the system, dry dichloroethane (100 mL) was pumped through the flow cell. The combined solutions were evaporated in vacuo. The residue was purified by column chromatography (SiO_2, dichloromethane/*n*-hexanes 2:1 to 1:1).

(*E*)-1,3-Diphenyl-2-propen-1-one (3a)[1]

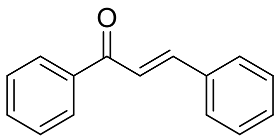

Isolated as a pale yellow solid. mp: 56–57 °C; ^{1}H NMR (400 MHz, $CDCl_3$): δ 8.05–8.01 (m, 2H), 7.82 (d, *J* = 15.7 Hz, 1H), 7.68–7.63 (m, 2H), 7.59 (tt, *J* = 1.4, 7.5 Hz, 1H), 7.54 (d, *J* = 15.7 Hz, 1H), 7.54–7.49 (m, 2H), 7.45–7.40 (m, 3H); ^{13}C NMR (100 MHz, $CDCl_3$): δ 190.53, 144.81, 138.20, 134.87, 132.73, 130.50, 128.92, 128.59, 128.47, 128.41, 122.10; MS (EI): *m/z* (%) = 208 (M^+, 18), 207 (M^+-H, 28), 178 (13), 131 (23), 103 (45), 77 (100).

(*E*)-1-Phenyl-3-*p*-tolylprop-2-en-1-one (3b)[2]

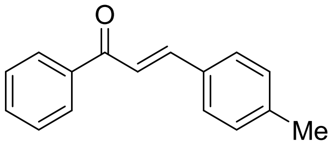

Isolated as a white solid. mp: 95-96 °C; ^{1}H NMR (400 MHz, $CDCl_3$): δ 7.93–7.88 (m, 2H), 7.69 (d, *J* = 15.7 Hz, 1H), 7.49–7.35 (m, 6H), 7.10 (d, *J* = 8.0 Hz, 2H), 2.27 (s, 3H); ^{13}C NMR (100 MHz, $CDCl_3$): δ 190.54, 144.88, 141.05, 138.34, 132.64, 132.14, 129.69, 128.57, 128.47, 128.45, 121.06, 21.52; MS (EI): *m/z* (%) = 222 (M^+, 12), 221 (M^+-H, 22), 207 (47), 178 (15), 145 (18), 115 (71), 105 (48), 91 (43), 77 (100), 65 (13).

(*E*)-3-(2,4-Dimethylphenyl)-1-phenylprop-2-en-1-one (3c)

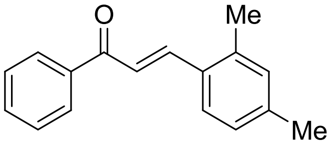

Isolated as a yellow oil. ^{1}H NMR (400 MHz, $CDCl_3$): δ 7.96 (d, *J* = 15.5 Hz, 1H), 7.96–7.85 (m, 2H), 7.46 (d, *J* = 7.7 Hz, 1H), 7.43–7.38 (m, 1H), 7.35–7.28 (m, 2H), 7.28 (d, *J* = 15.5 Hz, 1H), 6.92–6.86 (m, 2H), 2.28 (s, 3H), 2.17 (s, 3H); ^{13}C NMR (100 MHz, $CDCl_3$): δ 190.41, 142.36, 140.67, 138.39, 138.37, 132.65, 131.73, 131.02, 128.57, 128.45, 127.17, 126.42, 121.96, 21.36, 19.76; IR (neat): ν = 3059, 2920, 1661, 1596, 1498, 1447, 1330, 1215, 1016, 816, 777, 693 cm^{-1}; MS (EI): *m/z* (%) = 236 (M^+, 7), 221 (28), 129 (21), 115 (38), 105 (53), 91 (17), 77 (100).

(*E*)-1-Phenyl-3-*o*-tolylprop-2-en-1-one (3d)[3]

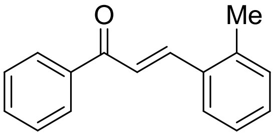

Isolated as a yellow oil. ^{1}H NMR (400 MHz, $CDCl_3$): δ 8.12 (d, *J* = 15.6 Hz, 1H), 8.05–8.01 (m, 2H), 7.69 (d, *J* = 6.6 Hz, 1H), 7.57 (tt, *J* = 1.4, 7.4 Hz, 1H), 7.52–7.48 (m, 2H), 7.46 (d, *J* = 15.6 Hz, 1H), 7.32–7.19 (m, 3H), 2.46 (s, 3H); ^{13}C NMR (100 MHz, $CDCl_3$): δ =190.32, 142.40, 138.33, 138.23, 133.90, 132.77, 130.91, 130.25, 128.61, 128.50, 126.41, 126.33, 123.10, 19.85; MS (EI): *m/z* (%) = 222 (M^+, 10), 207 (45), 178 (11), 115 (85), 105 (56), 91 (36), 77 (100).

(*E*)-3-(2-Methoxyphenyl)-1-phenylprop-2-en-1-one (3e)[4]

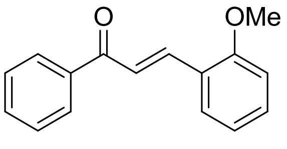

Isolated as a yellow oil. ^{1}H NMR (400 MHz, $CDCl_3$): δ 8.13 (d, *J* = 15.9 Hz, 1H), 8.05–8.00 (m, 2H), 7.65 (dd, *J* = 1.7, 7.7 Hz, 1H), 7.64 (d, *J* = 15.9 Hz, 1H), 7.59–7.54 (m, 1H), 7.51 (t, *J* = 7.1 Hz, 2H), 7.41–7.35 (m, 1H), 7.01 (t, *J* = 7.6 Hz, 1H), 6.95 (d, *J* = 8.2 Hz, 1H), 3.91 (s, 3H); ^{13}C NMR (100 MHz, $CDCl_3$): δ 191.08, 158.79, 140.37, 138.51, 132.51, 131.74, 129.20, 128.51, 128.50, 123.90, 122.85, 120.72, 111.23, 55.52; MS (EI): *m/z* (%) = 238 (M^+, 1), 207 (46), 131 (10), 118 (19), 105 (55), 89 (15), 77 (100), 63 (11).

(*E*)-3-(4-Methoxyphenyl)-1-phenylprop-2-en-1-one (3f)[5]

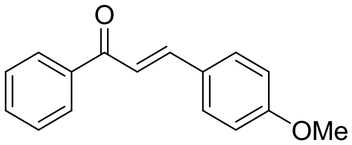

Isolated as a pale yellow solid. mp: 70-71 °C; ^{1}H NMR (300 MHz, $CDCl_3$): δ 8.05–7.97 (m, 2H), 7.79 (d, *J* = 15.7 Hz, 1H), 7.63–7.45 (m, 5H), 7.41 (d, *J* = 15.7 Hz, 1H), 6.98–6.88 (m, 2H), 3.84 (s, 3H); ^{13}C NMR (75 MHz, $CDCl_3$): δ 190.52, 161.67, 144.67, 138.50, 132.53, 130.22, 128.55, 128.40, 127.60, 119.76, 114.41, 55.39; MS (EI): *m/z* (%) = 238 (M^+, 32), 207 (16), 161 (23), 133 (22), 118 (34), 105 (41), 90 (25), 77 (100), 63 (22).

(*E*)-3-(2,4-Dimethoxyphenyl)-1-phenylprop-2-en-1-one (3g)

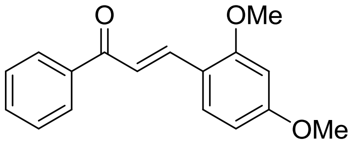

Isolated as a yellow oil. ^{1}H NMR (400 MHz, $CDCl_3$): δ 8.06 (d, *J* = 15.8 Hz, 1H), 8.03–7.98 (m, 2H), 7.57–7.45 (m, 5H), 6.53 (dd, *J* = 2.4, 8.6 Hz, 1H), 6.47 (d, *J* = 2.4 Hz, 1H), 3.89 (s, 3H), 3.85 (s, 3H); ^{13}C NMR (100 MHz, $CDCl_3$): δ 191.14, 163.01, 160.38, 140.49, 138.84, 132.24, 130.90, 128.43, 128.39, 120.40, 117.10, 105.41, 98.43, 55.52, 55.46; IR (neat): ν = 3060, 2941, 2838, 1655, 1592, 1458, 1299, 1211, 1029, 833,

780, 695 cm^{-1}; MS (EI): m/z (%) = 268 (M^+, 6), 237 (39), 105 (67), 77 (100).

(*E*)-3-(Benzo[*d*][1,3]dioxol-5-yl)-1-phenylprop-2-en-1-one (3h)

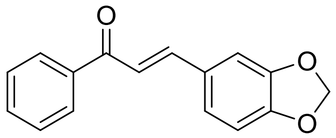

Isolated as a white solid. mp: 115-117 °C; ^{1}H NMR (300 MHz, $CDCl_3$): δ 8.03–7.97 (m, 2H), 7.73 (d, J = 15.6 Hz, 1H), 7.61–7.45 (m, 3H), 7.36 (d, J = 15.6 Hz, 1H), 7.17–7.08 (m, 2H), 6.83 (d, J = 8.0 Hz, 1H), 6.0 (s, 2H); ^{13}C NMR (75 MHz, $CDCl_3$): δ 190.31, 149.90, 148.39, 144.62, 138.37, 132.62, 129.33, 128.56, 128.39, 125.21, 120.06, 108.64, 106.63, 101.63; MS (EI): m/z (%) = 252 (M^+, 27), 251 (M^+-H, 19), 165 (27), 145 (25), 122 (24), 117 (33), 105 (55), 89 (65), 77 (100), 63 (18).

(*E*)-3-(Biphenyl-4-yl)-1-phenylprop-2-en-1-one (3i)[3]

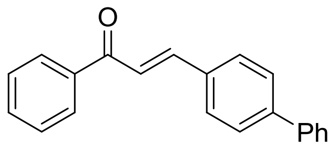

Isolated as a pale yellow solid. mp: 100-102 °C; ^{1}H NMR (400 MHz, $CDCl_3$): δ 8.10–8.04 (m, 2H), 7.88 (d, J = 15.7 Hz, 1H), 7.76-7.70 (m, 2H), 7.69–7.44 (m, 10H), 7.42–7.37 (m, 1 H); ^{13}C NMR (100 MHz, $CDCl_3$): δ 190.40, 144.35, 143.27, 140.09, 138.27, 133.84, 132.77, 128.98, 128.92, 128.63, 128.51, 127.91, 127.57, 127.04, 121.89; MS (EI): m/z (%) = 284 (M^+, 41), 283 (M^+-H, 32), 255 (12), 207 (41), 178 (61), 165 (12), 152 (13), 105 (53), 77 (100).

(*E*)-3-(2-Bromophenyl)-1-phenylprop-2-en-1-one (3j)[6]

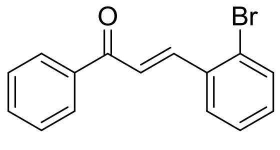

Isolated as a pale yellow oil. ^{1}H NMR (400 MHz, $CDCl_3$): δ 8.13 (d, J = 15.7 Hz, 1H), 8.05–7.99 (m, 2H), 7.72 (dd, J = 1.6, 7.8 Hz, 1H), 7.64-7.54 (m, 2H), 7.53–7.46 (m, 2H), 7.42 (d, J = 15.7 Hz, 1H), 7.34 (t, J = 7.3 Hz, 1H), 7.22 (td, J = 1.7, 7.8 Hz, 1H); ^{13}C NMR (100 MHz, $CDCl_3$): δ 190.26, 143.08, 137.84, 134.99,

133.51, 132.94, 131.33, 128.65, 128.61, 127.87, 127.72, 125.86, 124.96; MS (EI): *m/z* (%) = 288 (M^{+}+2, 1), 286 (M^{+}, 1), 207 (46), 178 (14), 105 (47), 102 (37), 77 (100).

(*E*)-3-(2-Chlorophenyl)-1-phenylprop-2-en-1-one (3k)[7]

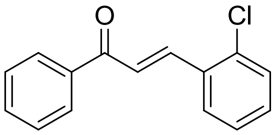

Isolated as a yellow oil. ^{1}H NMR (400 MHz, $CDCl_3$): δ 8.04 (d, *J* = 15.8 Hz, 1H), 7.90–7.85 (m, 2H), 7.82–7.57 (m, 1H), 7.44 (tt, *J* = 1.3, 7.9 Hz, 1H), 7.38–7.31 (m, 3H), 7.29–7.24 (m, 1H), 7.12–7.18 (m, 2H); ^{13}C NMR (100 MHz, $CDCl_3$): δ 190.30, 140.52, 137.84, 135.43, 133.15, 132.95, 131.19, 130.24, 128.65, 128.59, 127.78, 127.10, 124.66; MS (EI): *m/z* (%) = 242 (M^{+}, 4), 207 (37), 178 (9), 137 (9), 105 (38), 102 (24), 77 (100).

(*E*)-3-(3-Chlorophenyl)-1-phenylprop-2-en-1-one (3l)[8]

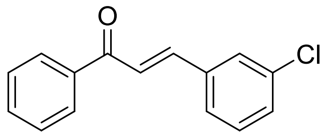

Isolated as a pale yellow solid. mp: 72–73 °C; ^{1}H NMR (400 MHz, $CDCl_3$): δ 8.04–7.99 (m, 2H), 7.71 (d, *J* = 15.7 Hz, 1H), 7.62–7.55 (m, 2H), 7.51 (d, *J* = 15.7 Hz, 1H), 7.51–7.46 (m, 3H), 7.38–7.29 (m, 2H); ^{13}C NMR (100 MHz, $CDCl_3$): δ 189.91, 142.92, 137.85, 136.70, 134.92, 132.98, 130.28, 130.17, 128.66, 128.51, 127.88, 126.75, 123.17; MS (EI): *m/z* (%) = 242 (M^{+}, 11), 241 (M^{+}-H, 15), 207 (18), 178 (12), 165 (9), 137 (14), 105 (50), 102 (41), 77 (100).

(*E*)-3-Phenyl-1-*p*-tolylprop-2-en-1-one (3m)[9]

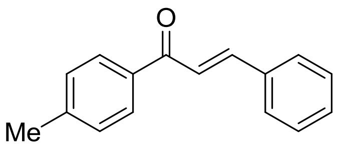

Isolated as a white solid. mp: 53–54 °C; ^{1}H NMR (400 MHz, $CDCl_3$): δ 7.95 (dt, *J* = 1.8, 8.2 Hz, 2H), 7.82 (d, *J* = 15.7 Hz, 1H), 7.67–7.62 (m, 2H), 7.54 (d, *J* = 15.7 Hz, 1H), 7.45–7.38 (m, 3H), 7.30 (d, *J* = 8.5 Hz, 2H), 2.43 (s, 3H); ^{13}C NMR (100 MHz, $CDCl_3$): δ 189.89, 144.31, 143.60, 135.62, 134.99, 130.39, 129.32, 128.91, 128.64, 128.39, 127.07, 21.66; MS (EI): *m/z*

(%) = 222 (M^+, 27), 221 (M^+-H, 32), 178 (14), 131 (16), 119 (40), 103 (53), 91 (100), 77 (42), 65 (32).

(*E*)-1-(4-Ethylphenyl)-3-phenylprop-2-en-1-one (3n)[9]

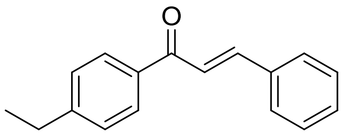

Isolated as a white solid. mp: 57-58 °C; ^{1}H NMR (400 MHz, $CDCl_3$): δ 7.99 (d, *J* = 8.2 Hz, 2H), 7.83 (d, *J* = 15.7 Hz, 1H), 7.72-7.61 (m, 2H), 7.56 (d, *J* = 15.7 Hz, 1H), 7.49-7.38 (m, 3H), 7.34 (d, *J* = 8.0 Hz, 2H), 2.75 (q, *J* = 7.6 Hz, 2H), 1.30 (t, *J* = 7.6 Hz, 3H); ^{13}C NMR (100 MHz, $CDCl_3$): δ 189.99, 149.77, 144.33, 135.85, 135.00, 130.38, 128.91, 128.73, 128.37, 128.12, 122.13, 28.96, 15.21; MS (EI): *m*/*z* (%) = 236 (M^+, 50), 235 (M^+-H, 48), 207 (36), 178 (22), 133 (59), 103 (100), 89 (37), 77 (79).

(*E*)-1-(4-Pentylphenyl)-3-phenylprop-2-en-1-one (3o)

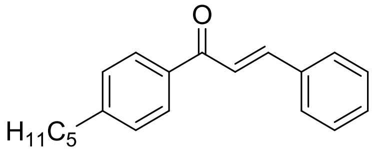

Isolated as a white solid. mp: 50-51 °C; ^{1}H NMR (400 MHz, $CDCl_3$): δ 7.97 (dt, *J* = 1.8, 8.2 Hz, 2H), 7.82 (d, *J* = 15.7 Hz, 1H), 7.68-7.62 (m, 2H), 7.55 (d, *J* = 15.7 Hz, 1H), 7.45-7.39 (m, 3H), 7.32 (d, *J* = 8.1 Hz, 2H), 2.69 (t, *J* = 7.7 Hz, 2H), 1.67 (q, *J* = 7.5 Hz, 2H), 1.42-1.29 (m, 4H), 0.91 (t, *J* = 7.0 Hz, 3H); ^{13}C NMR (100 MHz, $CDCl_3$): δ 189.98, 148.56, 144.30, 135.83, 135.01, 130.37, 128.90, 128.66, 128.64, 128.37, 122.12, 35.98, 31.43, 30.79, 22.49, 13.99; IR (KBr): *ν* = 3063, 2925, 2855, 1654, 1594, 1451, 1333, 1177, 991, 847, 757, 674 cm^{-1}; MS (EI): *m*/*z* (%) = 278 (M^+, 65), 277 (100), 221 (34), 207 (65), 193 (62), 178 (41), 131 (82), 103 (92), 91 (89), 77 (42).

(*E*)-1-(4-*tert*-Butylphenyl)-3-phenylprop-2-en-1-one (3p)[9]

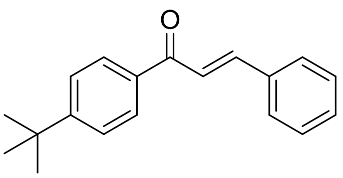

Isolated as a white solid. mp: 92-94 °C; ^{1}H NMR (400 MHz, $CDCl_3$): δ 8.00 (dt, *J* =

2.1, 8.2 Hz, 2H), 7.83 (d, *J* = 15.7 Hz, 1H), 7.68–7.64 (m, 2H), 7.57 (d, *J* = 15.7 Hz, 1H), 7.53 (dt, *J* = 2.1, 8.2 Hz, 2H), 3.45–3.39 (m, 3H), 1.38 (s, 9H); ^{13}C NMR (100 MHz, $CDCl_3$): δ 189.99, 156.53, 144.33, 135.57, 135.01, 130.39, 128.92, 128.49, 128.39, 125.57, 122.16, 35.11, 31.11; MS (EI): *m*/*z* (%) = 264 (M^+, 25), 263 (M^+-H, 37), 249 (20), 207 (32), 131 (62), 115 (28), 103 (100), 91 (44), 77 (37).

(*E*)-1-(4-Methoxyphenyl)-3-phenylprop-2-en-1-one (3q)[9]

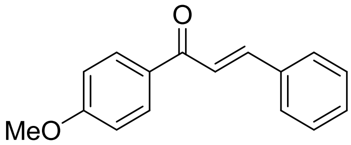

Isolated as a pale yellow solid. mp: 45-47 °C; ^{1}H NMR (300 MHz, $CDCl_3$): δ 7.94 (d, *J* = 8.4 Hz, 2H), 7.81 (d, *J* = 15.7 Hz, 1H), 7.69–7.62 (m, 2H), 7.54 (d, *J* = 15.7 Hz, 1H), 7.46–7.38 (m, 3H), 7.31 (d, *J* = 8.5 Hz, 2H), 2.44 (s, 3H); ^{13}C NMR (75 MHz, $CDCl_3$): δ 190.00, 144.37, 143.62, 135.62, 134.99, 130.39, 129.31, 128.91, 128.64, 128.38, 122.10, 21.67; MS (EI): *m*/*z* (%) = 238 (M^+, 4), 221 (39), 178 (22), 131 (25), 119 (47), 103 (58), 91 (100), 77 (62), 65 (36).

(*E*)-1,3-Bis(4-methoxyphenyl)prop-2-en-1-one (3r)[10]

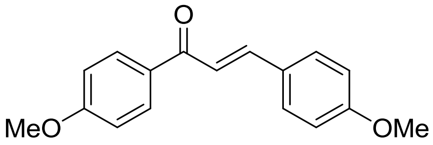

Isolated as a pale yellow solid. mp: 94-96 °C; ^{1}H NMR (400 MHz, $CDCl_3$): δ 8.04 (d, *J* = 8.0 Hz, 2H), 7.79 (d, *J* = 15.6 Hz, 1H), 7.61 (d, *J* = 8.0 Hz, 2H), 7.44 (d, *J* = 15.6 Hz, 1H), 6.99 (d, *J* = 12.0 Hz, 2H), 6.94 (d, *J* = 8.0 Hz, 2H), 3.89 (s, 3H), 3.86 (s, 3H); ^{13}C NMR (100 MHz, $CDCl_3$): δ 188.72, 163.23, 161.47, 143.76, 131.33, 130.65, 130.06, 127.79, 119.54, 114.35, 113.75, 55.44, 55.36; MS (EI): *m*/*z* (%) = 269 (M^++H, 17), 268 (M^+, 49), 267 (M^+-H, 27), 253 (16), 225 (16), 161 (22), 135 (100), 118 (14), 107 (24), 92 (46), 77 (59), 63 (33).

(*E*)-1-(4-Fluorophenyl)-3-phenylprop-2-en-1-one (3s)[9]

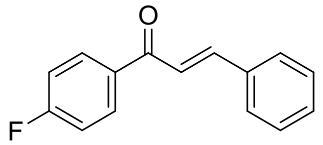

Isolated as a white solid. mp: 78–79 °C; ^{1}H NMR (400 MHz, $CDCl_3$): δ 8.07 (dd, *J* = 5.4, 8.8 Hz, 2H), 7.83 (d, *J* = 15.7 Hz, 1H), 7.70–7.60 (m, 2H), 7.51 (d, *J* = 15.7 Hz, 1H), 7.47–7.36 (m, 3H), 7.18 (t, *J* = 8.6 Hz, 2H); ^{13}C NMR (100 MHz, $CDCl_3$): δ 188.74, 165.58 (d, *J* = 253.1 Hz, CF), 144.99, 134.74, 134.50 (d, *J* = 3.0 Hz, 1C), 131.06 (d, *J* = 9.2 Hz, 2CH), 130.62, 128.95, 128.44, 121.56, 115.70 (d, *J* = 21.7 Hz, 2CH); ^{19}F NMR (376 MHz, $CDCl_3$): δ −105.59 (1F); MS (EI): *m/z* (%) = 226 (M^+, 22), 225 (M^+-H, 48), 196 (16), 131 (25), 123 (68), 103 (87), 95 (100), 77 (62), 63 (21).

(*E*)-3-(Benzo[*d*][1,3]dioxol-5-yl)-1-(4-fluorophenyl)prop-2-en-1-one (3t)

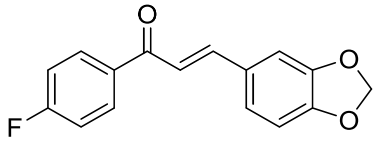

Isolated as a pale yellow solid. mp: 134–135 °C; ^{1}H NMR (300 MHz, $CDCl_3$): δ 8.05 (dd, *J* = 5.4, 9.0 Hz, 2H), 7.75 (d, *J* = 15.5 Hz, 1H), 7.34 (d, *J* = 15.5 Hz, 1H), 7.23–7.09 (m, 4H), 6.85 (d, *J* = 8.0 Hz, 1H), 6.04 (s, 2H); ^{13}C NMR (75 MHz, $CDCl_3$): δ 188.64, 165.50 (d, *J* = 254.0 Hz, CF), 149.99, 148.42, 144.86, 134.70, 130.95 (d, *J* = 9.2 Hz, 1CH), 129.21, 125.29, 119.55, 115.66 (d, *J* = 21.8 Hz, 1CH), 108.67, 106.61, 101.65; ^{19}F NMR (282 MHz, $CDCl_3$): δ −105.90 (1F); IR (KBr): ν = 3066, 2915, 1662, 1596, 1493, 1449, 1228, 1031, 975, 924, 794 cm^{-1}; MS (EI): *m/z* (%) = 270 (M^+, 43), 269 (M^+-H, 30), 183 (39), 145 (32), 123 (88), 117 (41), 95 (100), 89 (75), 75 (43), 63 (31).

(*E*)-1-Cyclohexyl-3-phenylprop-2-en-1-one (3u)[11]

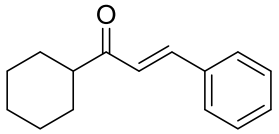

Isolated as a brown solid. mp: 51-53 °C; ^{1}H NMR (400 MHz, $CDCl_3$): δ 7.61 (d, *J* = 16.0 Hz, 1H), 7.59–7.56 (m, 2H), 7.42–7.36 (m, 3H), 6.83 (d, *J* = 16.0 Hz, 1H), 2.67 (tt, *J* = 3.4, 11.4 Hz, 1H), 1.94–1.82

(m, 4H), 1.78–1.65 (m, 1H), 1.50–1.23 (m, 5H); ^{13}C NMR (100 MHz, $CDCl_3$): δ 203.07, 142.17, 134.73, 130.24, 128.85, 128.22, 124.70, 49.38, 28.70, 25.89, 25.74; MS (EI): *m/z* (%) = 214 (M^+, 7), 131(100), 103 (65), 77 (28).

References

1. D.-N. Liu, S.-K. Tian, *Chem. Eur. J.* **2009**, *15*, 4538–4542.
2. L.-J. Zhang, Y.-Z. Huang, *J. Organomet. Chem.* **1993**, *454*, 101–103.
3. J. Hine, M. J. Skoglund, *J. Org. Chem*. **1982**, *47*, 4758–4766.
4. K. V. Sashidhara, J. N. Rosaiah, A. Kumar, *Synth. Commun.* **2009**, *39*, 2288–2296.
5. B. C. Ranu, R. Jana, *J. Org. Chem.* **2005**, *70*, 8621–8624.
6. C. Peppe, R. P. das Chagas, *J. Organomet. Chem*. **2006**, *691*, 5856–5860.
7. D. Huang, J.-X. Wang, Y. Hu, Y. Zhang, J. Tang, *Synth. Commun.* **2002**, *32*, 971–979.
8. A. Stroba, *J. Med. Chem*. **2009**, *52*, 4683–4693.
9. X.-F. Wu, H. Neumann, A. Spannenberg, T. Schulz, H. Jiao, M. Beller, *J. Am. Chem. Soc*. **2010**, *132*, 14596–14602.
10. M. D. Bowman, M. M. Jacobson, H. E. Blackwell, *Org. Lett*. **2006**, *8*, 1645–1648.
11. R. J. Cox, A. S. Evitt. *Org. Biomol. Chem.*, **2007**, *5*, 229–232.

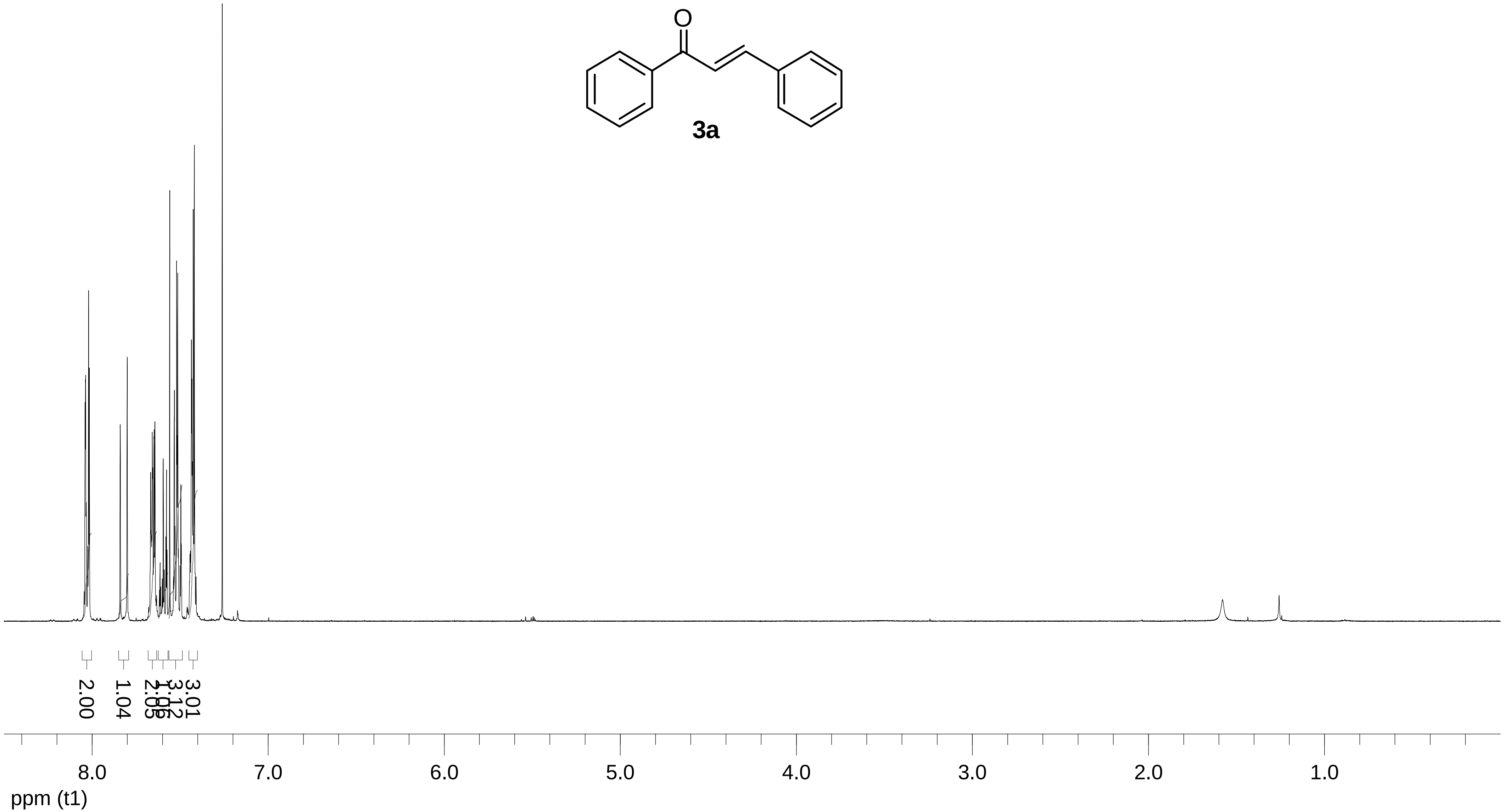
O
3a
2.00
1.04
2.05
1.06
3.12
3.01
8.0
7.0
6.0
5.0
4.0
3.0
2.0
1.0
ppm (t1)

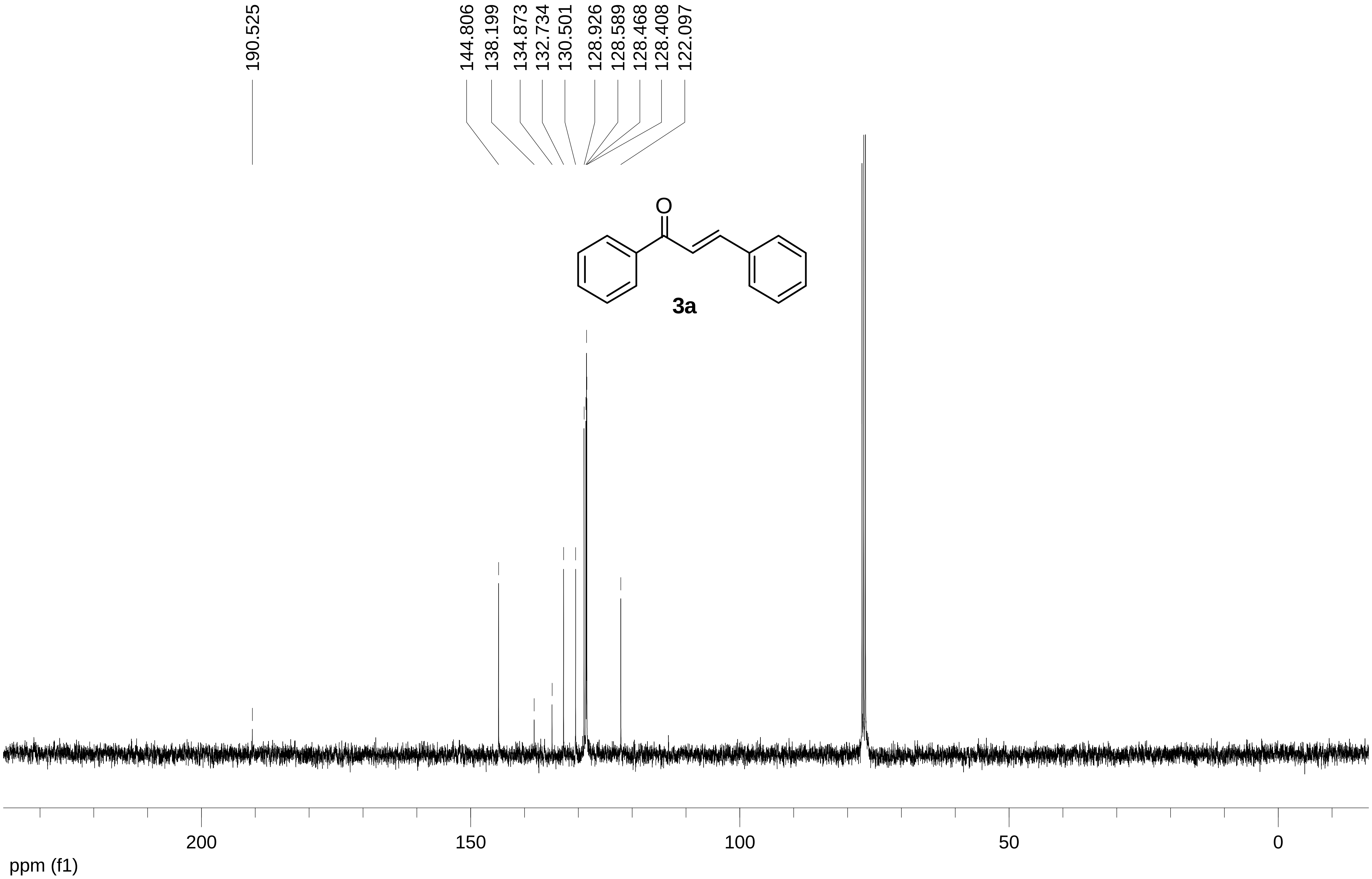
190.525
144.806
138.199
134.873
132.734
130.501
128.926
128.589
128.468
128.408
122.097
O
3a
200
150
100
50
0
ppm (f1)

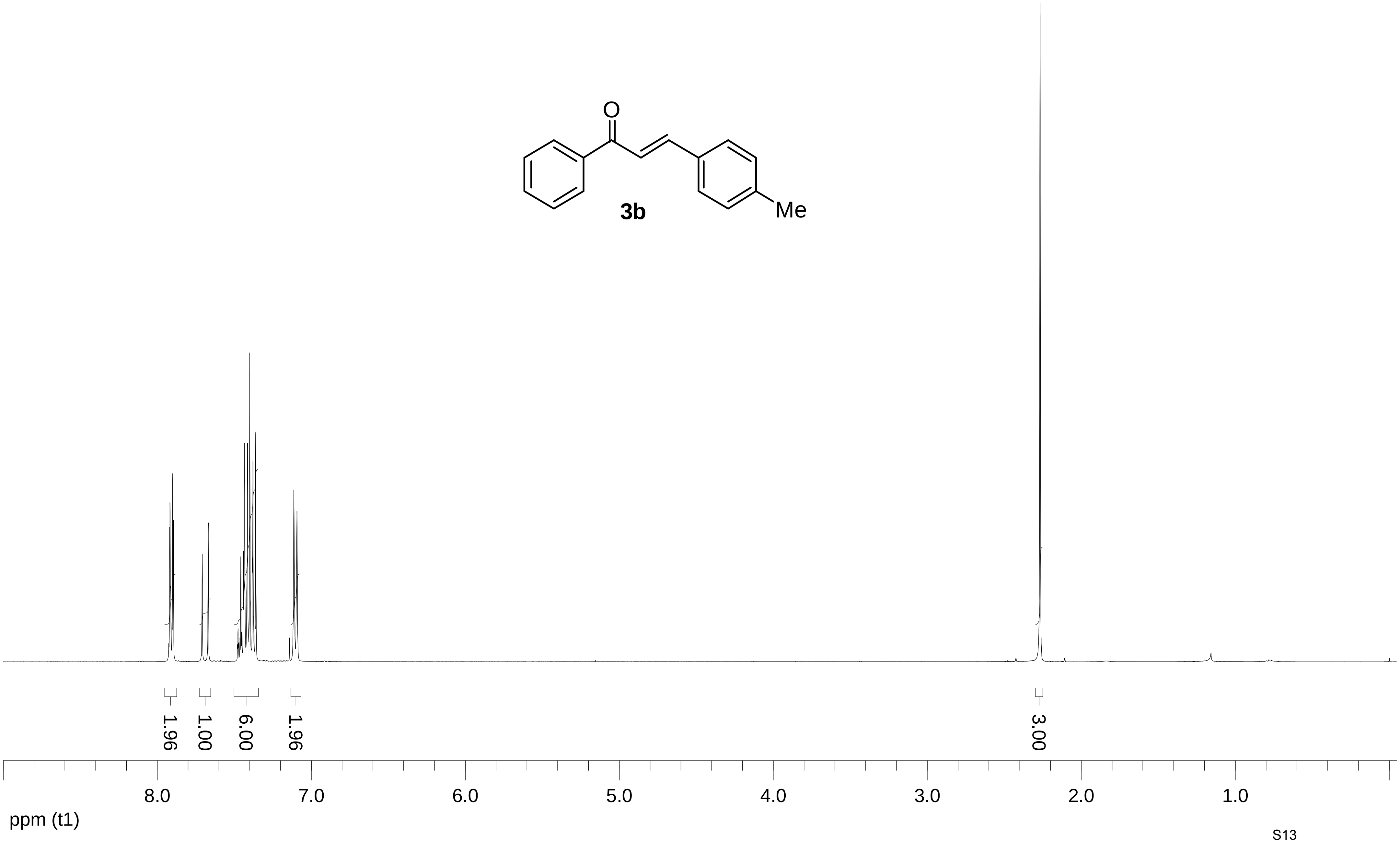

O
3b
Me
1.96
1.00
6.00
1.96
3.00
8.0
7.0
6.0
5.0
4.0
3.0
2.0
1.0
ppm (t1)

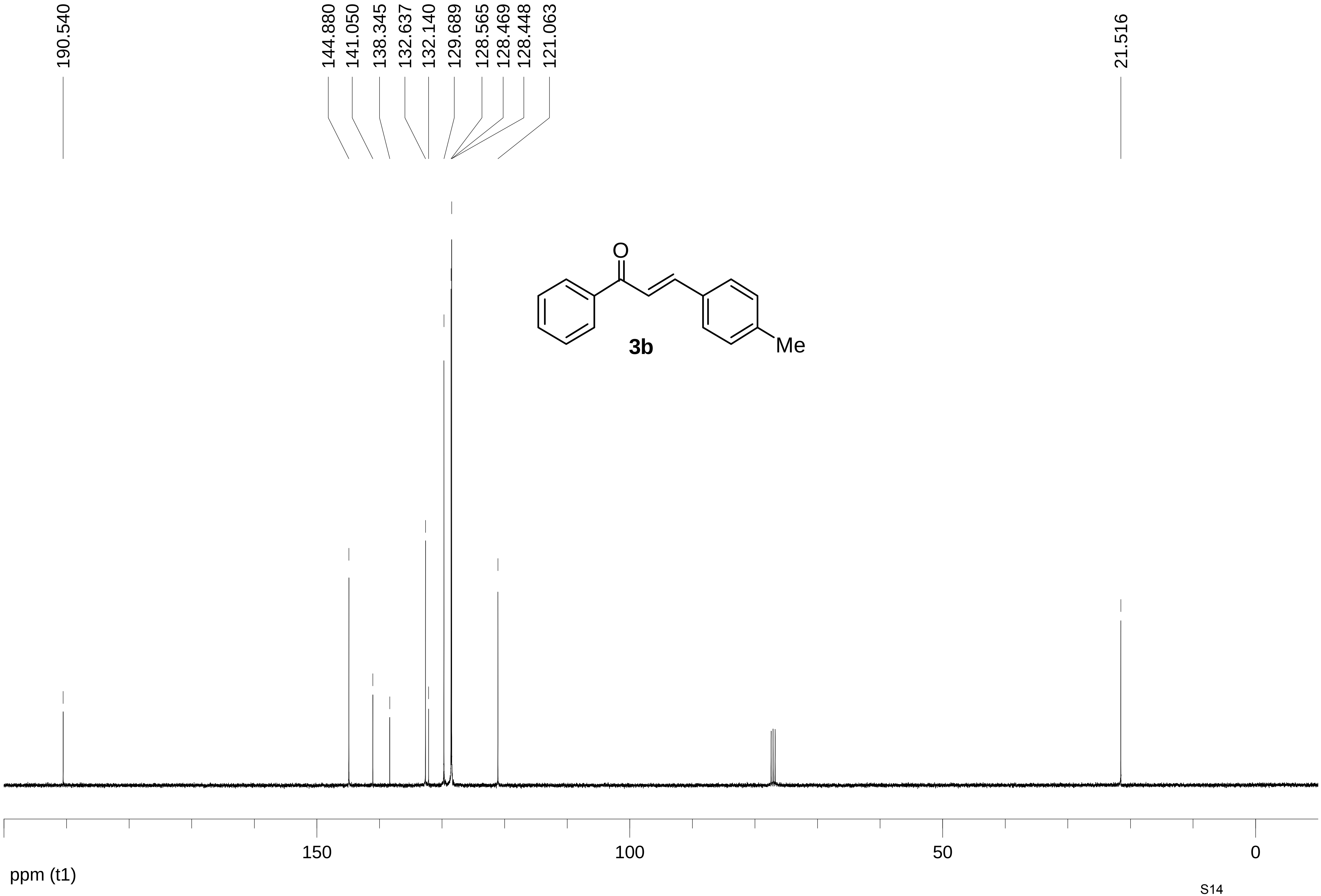
190.540
144.880
141.050
138.345
132.637
132.140
129.689
128.565
128.469
128.448
121.063
21.516
O
3b
Me
150
100
50
0
ppm (t1)

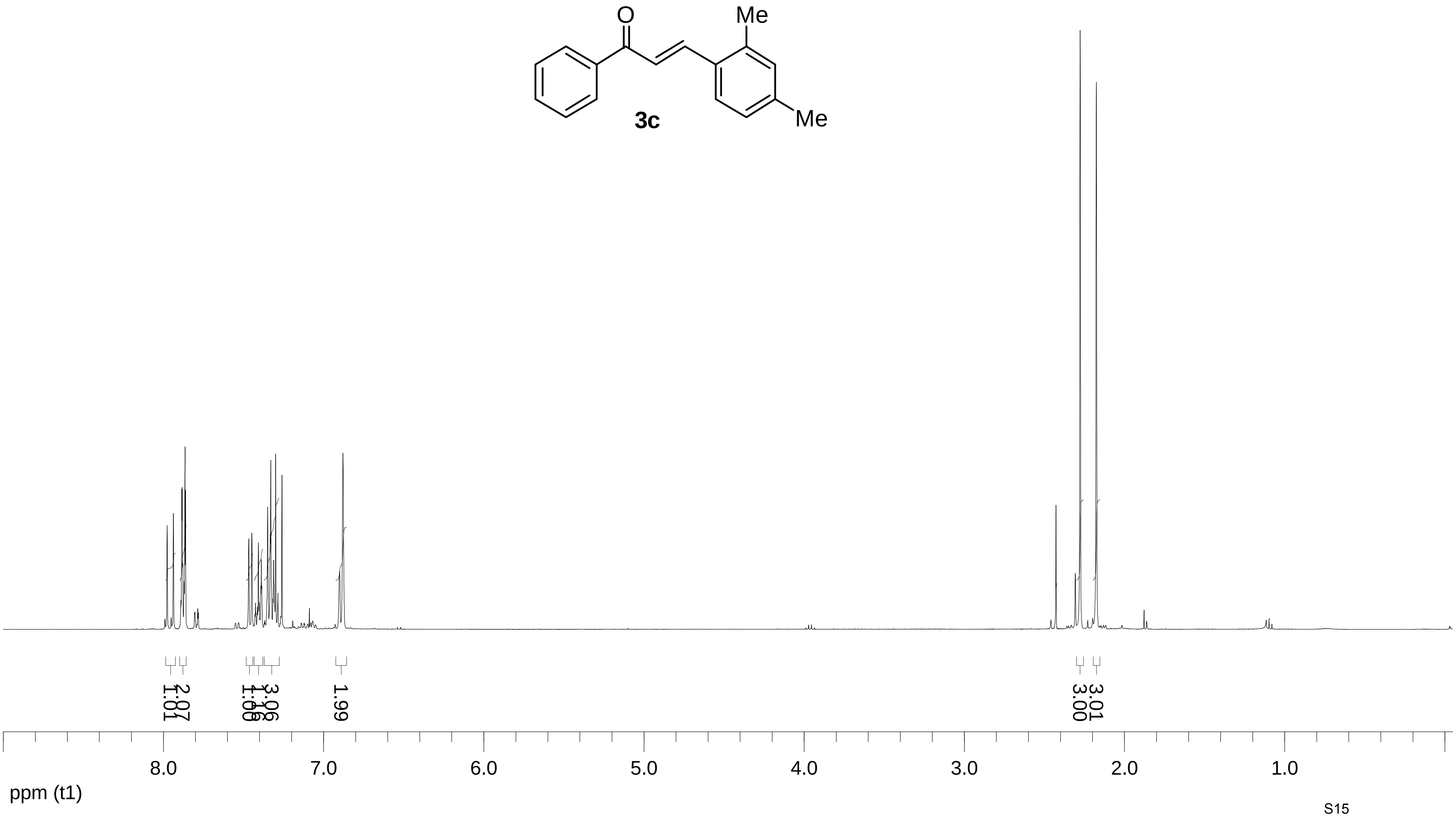
O
Me
Me
3c
1.01
2.07
3.06
1.99
3.00
3.01
8.0
7.0
6.0
5.0
4.0
3.0
2.0
1.0
ppm (t1)

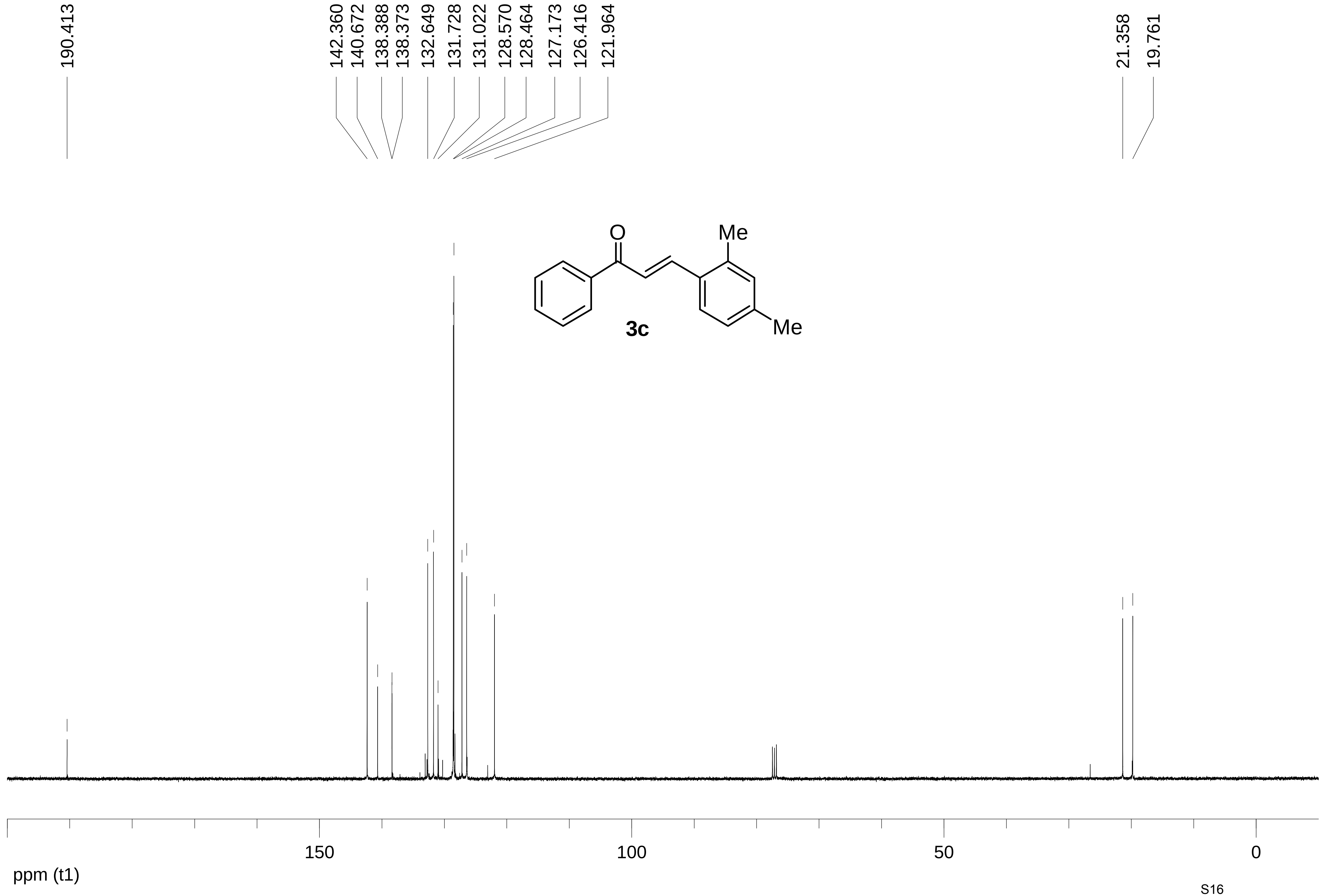
190.413
142.360
140.672
138.388
138.373
132.649
131.728
131.022
128.570
128.464
127.173
126.416
121.964
21.358
19.761
O
Me
Me
3c
150
100
50
0
ppm (t1)

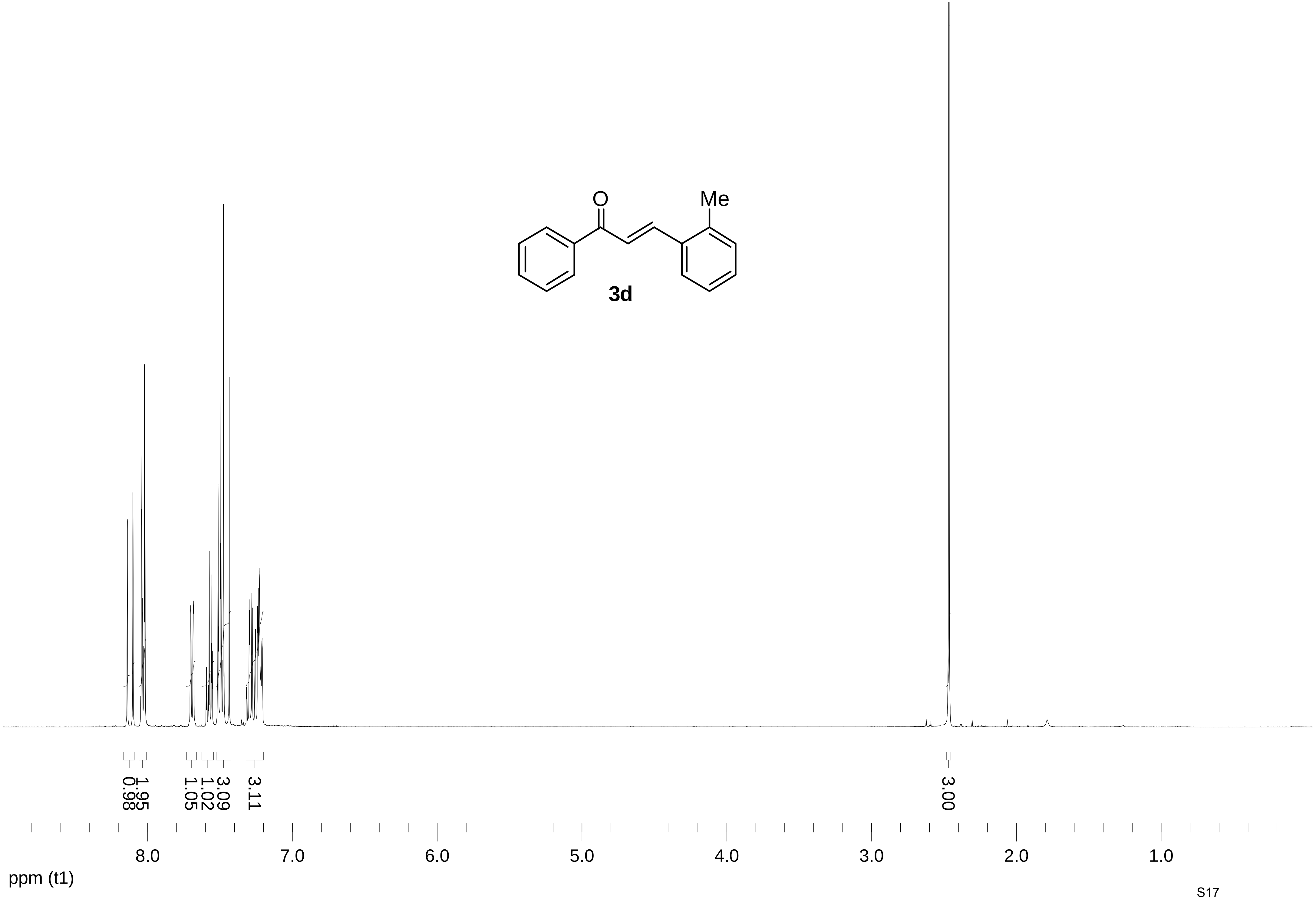
O
Me
3d
0.98
1.95
1.05
1.02
3.09
3.11
3.00
8.0
7.0
6.0
5.0
4.0
3.0
2.0
1.0
ppm (t1)

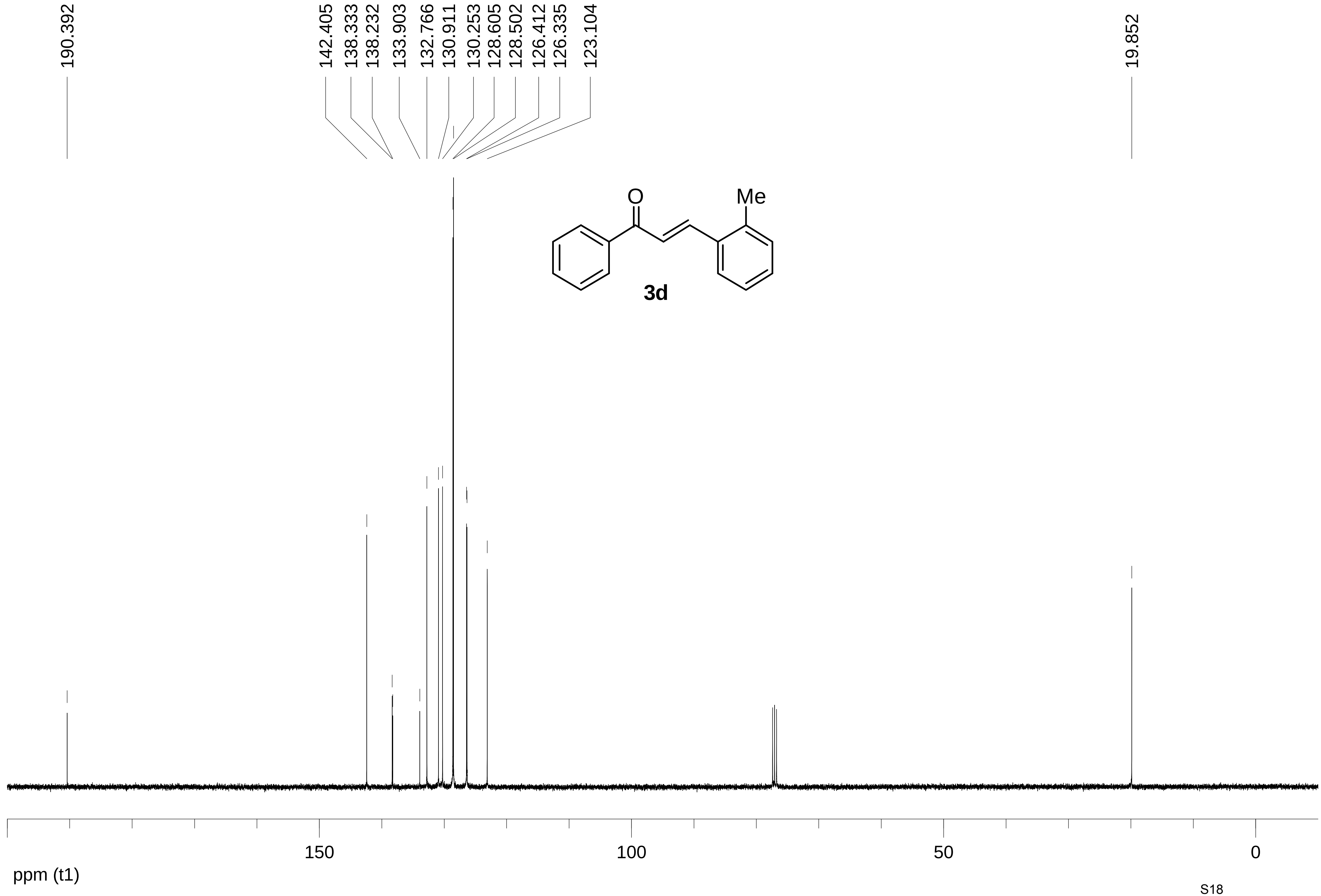

190.392
142.405
138.333
138.232
133.903
132.766
130.911
130.253
128.605
128.502
126.412
126.335
123.104
19.852
O
Me
3d
150
100
50
0
ppm (t1)

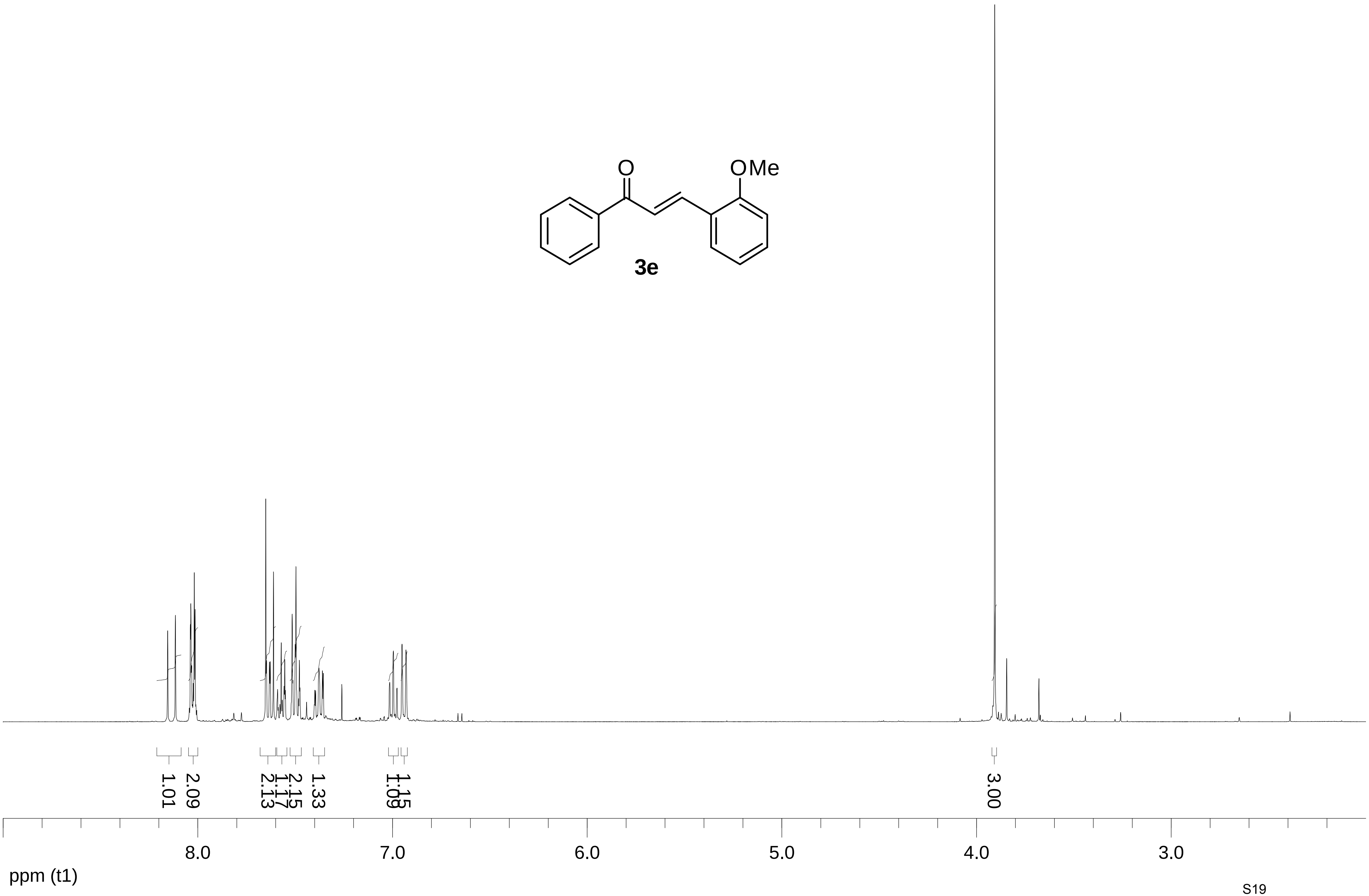
O
OMe
3e
1.01
2.09
2.13
1.17
2.15
1.33
1.09
1.15
3.00
8.0
7.0
6.0
5.0
4.0
3.0
ppm (t1)

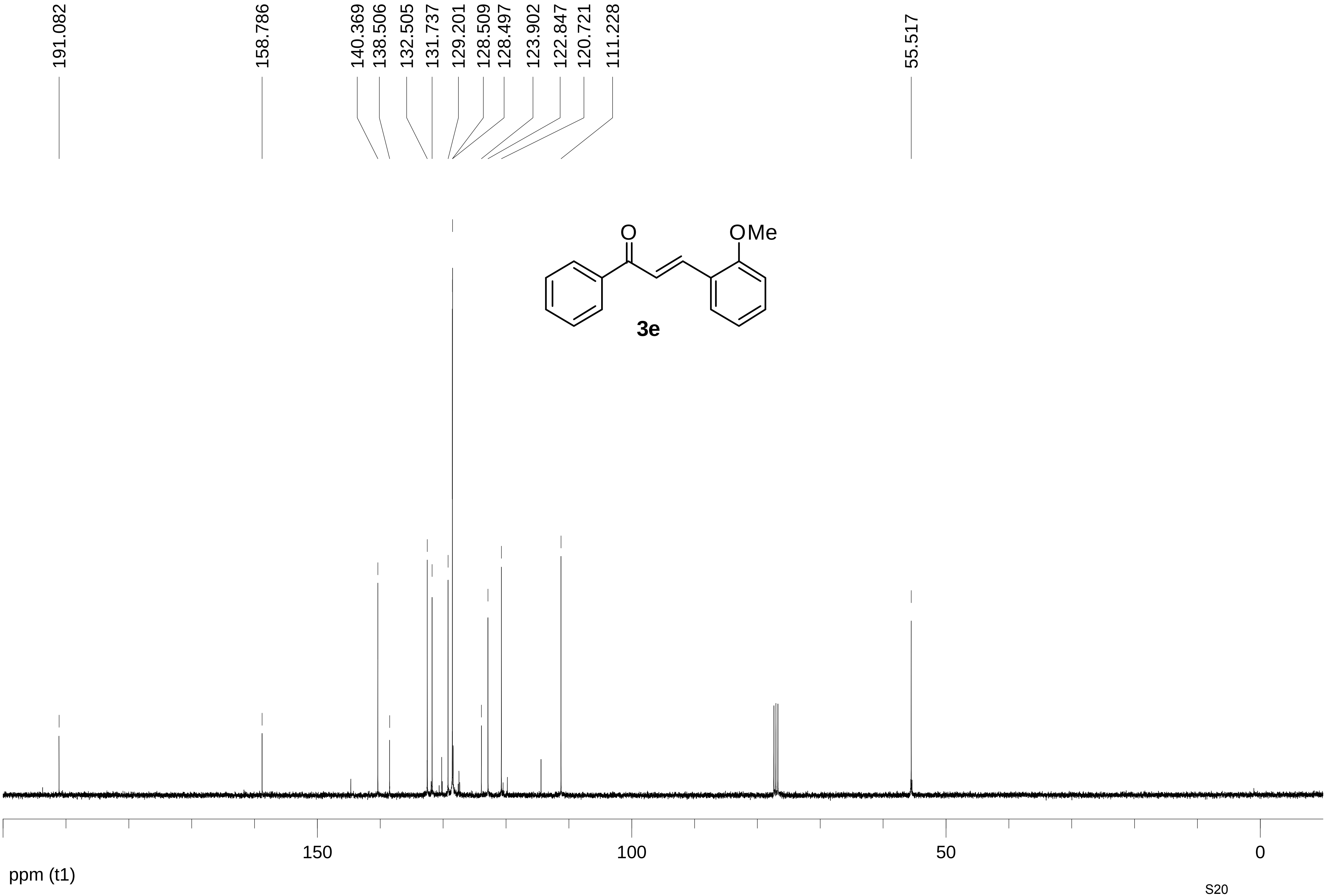
191.082
158.786
140.369
138.506
132.505
131.737
129.201
128.509
128.497
123.902
122.847
120.721
111.228
55.517
O
OMe
3e
150
100
50
0
ppm (t1)

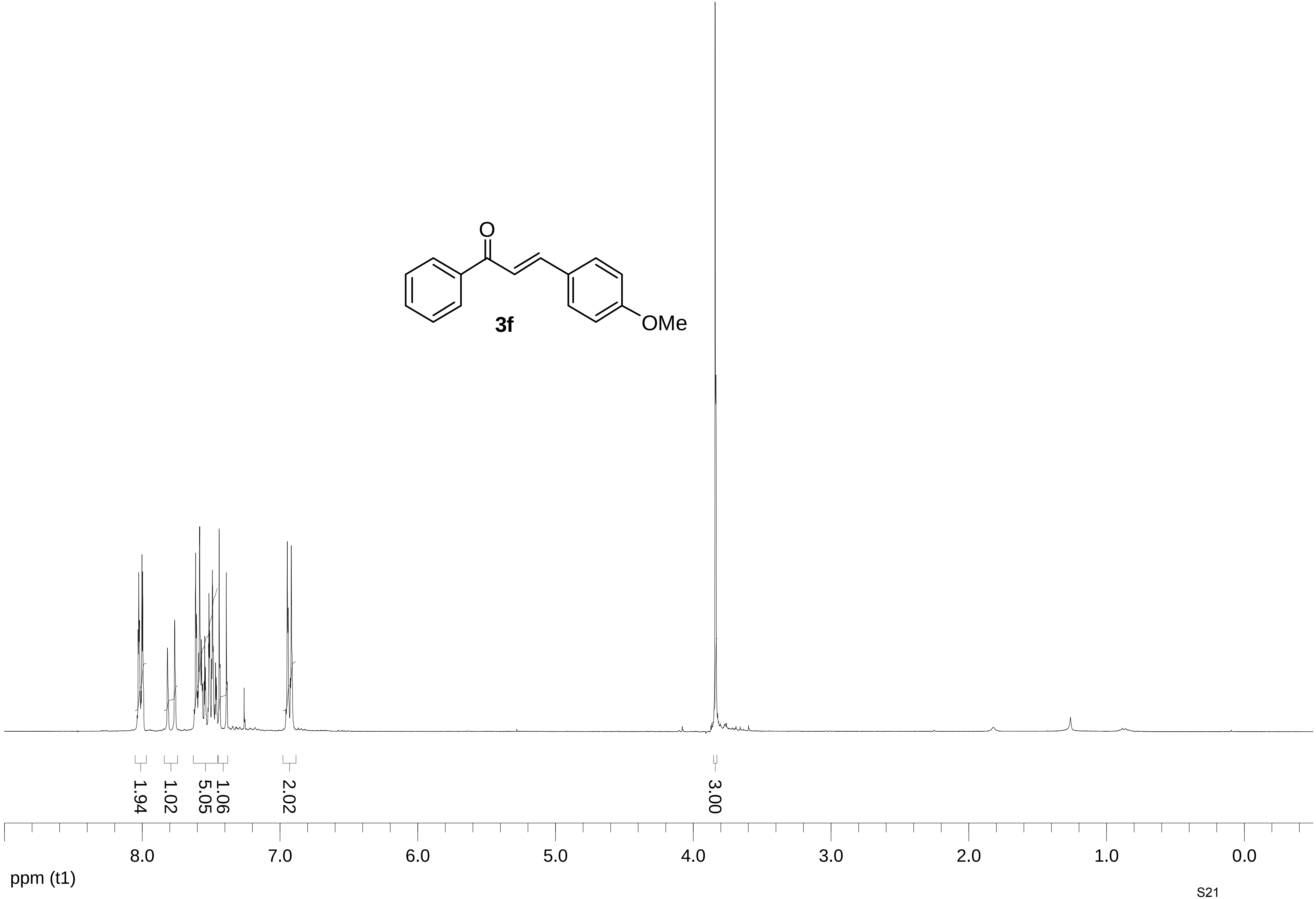
O
3f
OMe
1.94
1.02
5.05
1.06
2.02
3.00
8.0
7.0
6.0
5.0
4.0
3.0
2.0
1.0
0.0
ppm (t1)

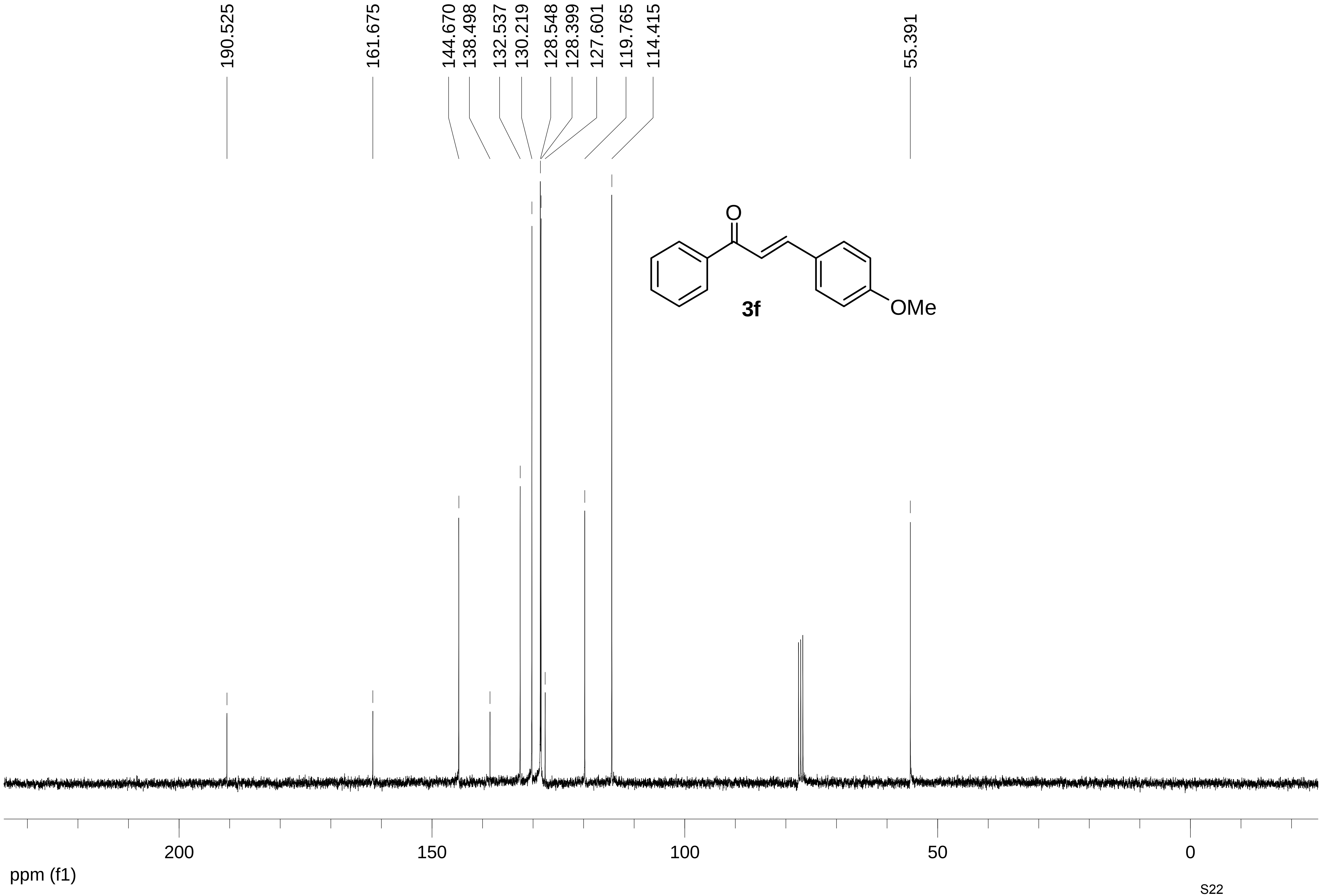
190.525
161.675
144.670
138.498
132.537
130.219
128.548
128.399
127.601
119.765
114.415
55.391
O
OMe
3f
200
150
100
50
0
ppm (f1)

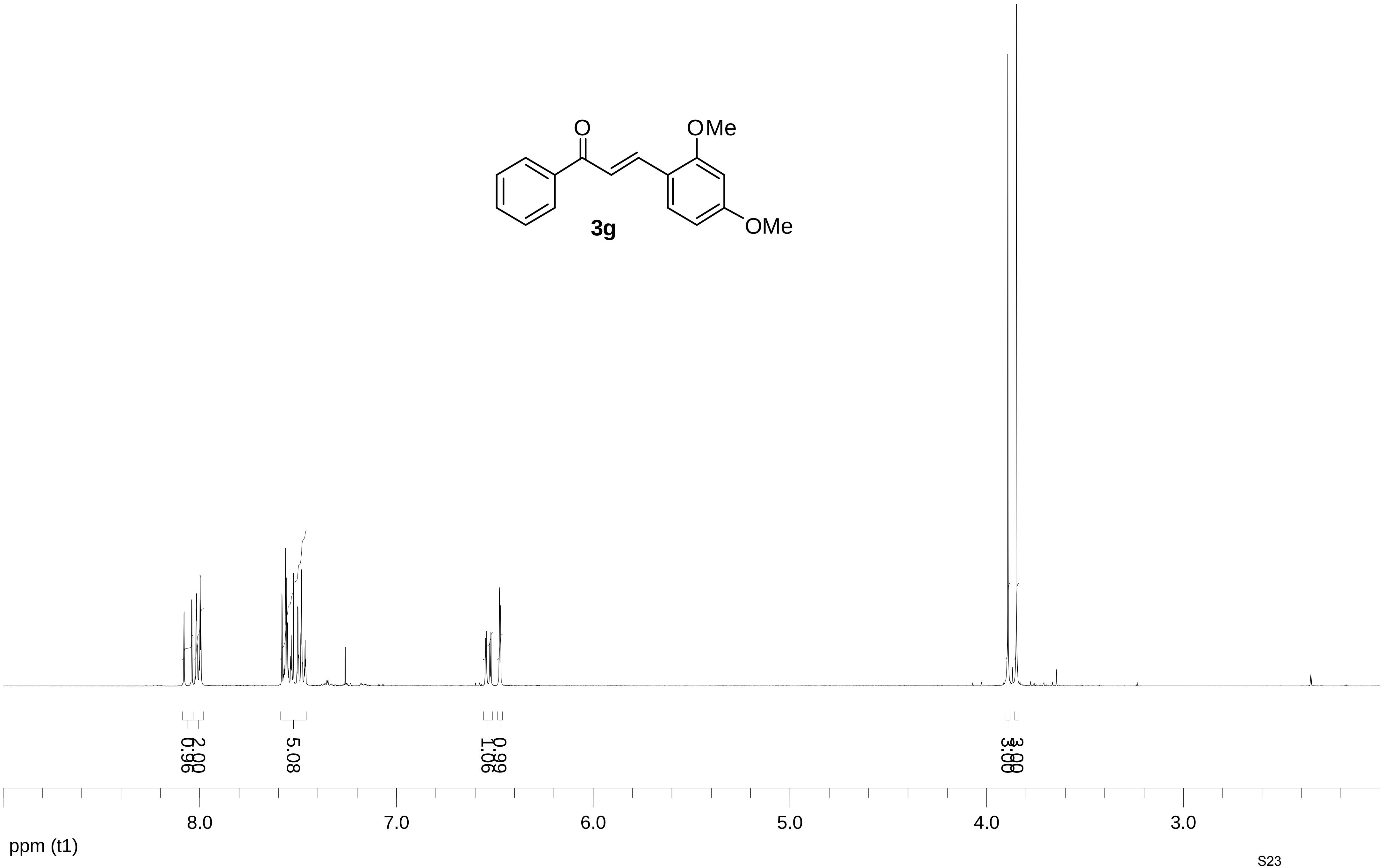
O
OMe
OMe
3g
2.00
5.08
0.99
3.00
8.0
7.0
6.0
5.0
4.0
3.0
ppm (t1)

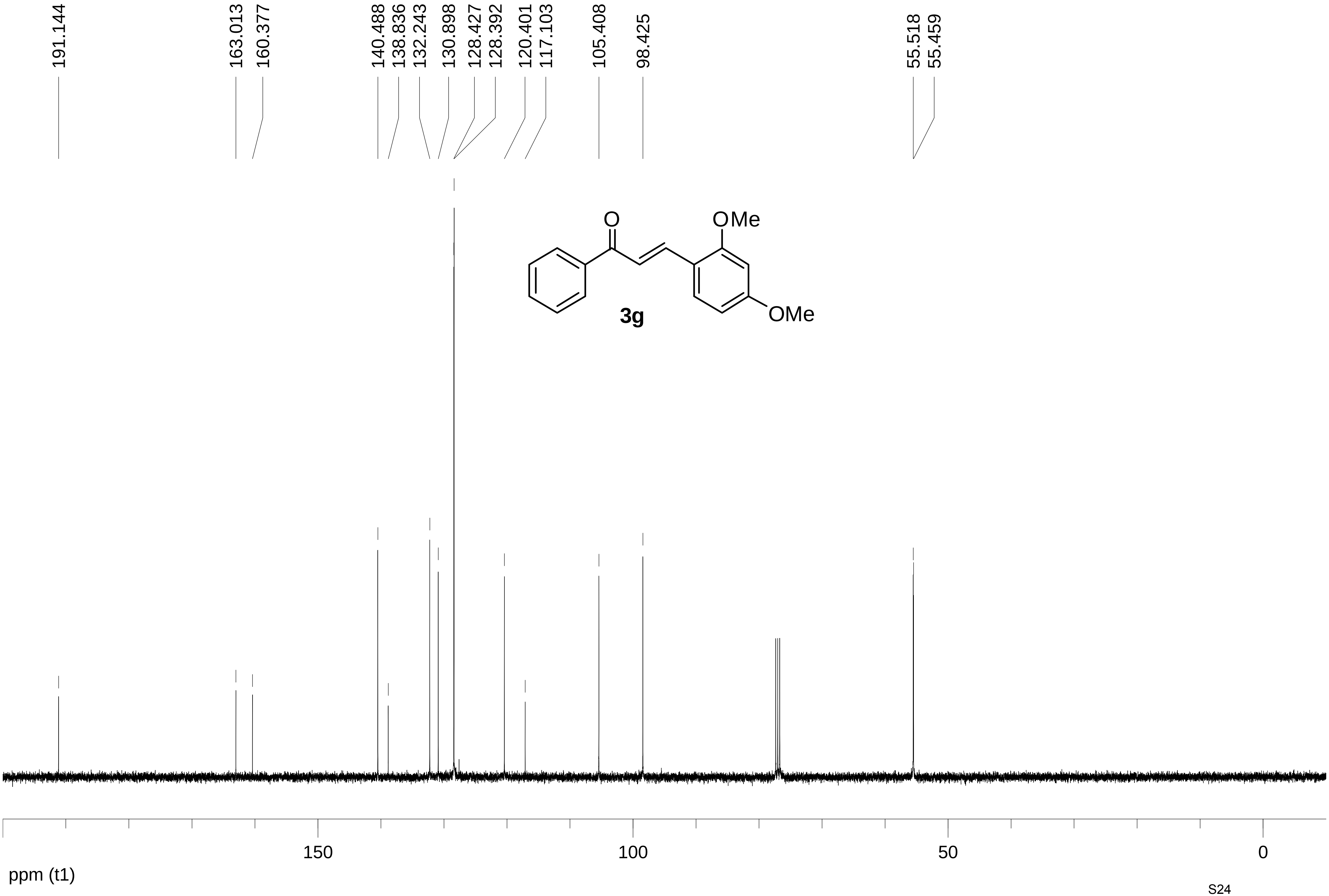
191.144
163.013
160.377
140.488
138.836
132.243
130.898
128.427
128.392
120.401
117.103
105.408
98.425
55.518
55.459
O
OMe
OMe
3g
150
100
50
0
ppm (t1)

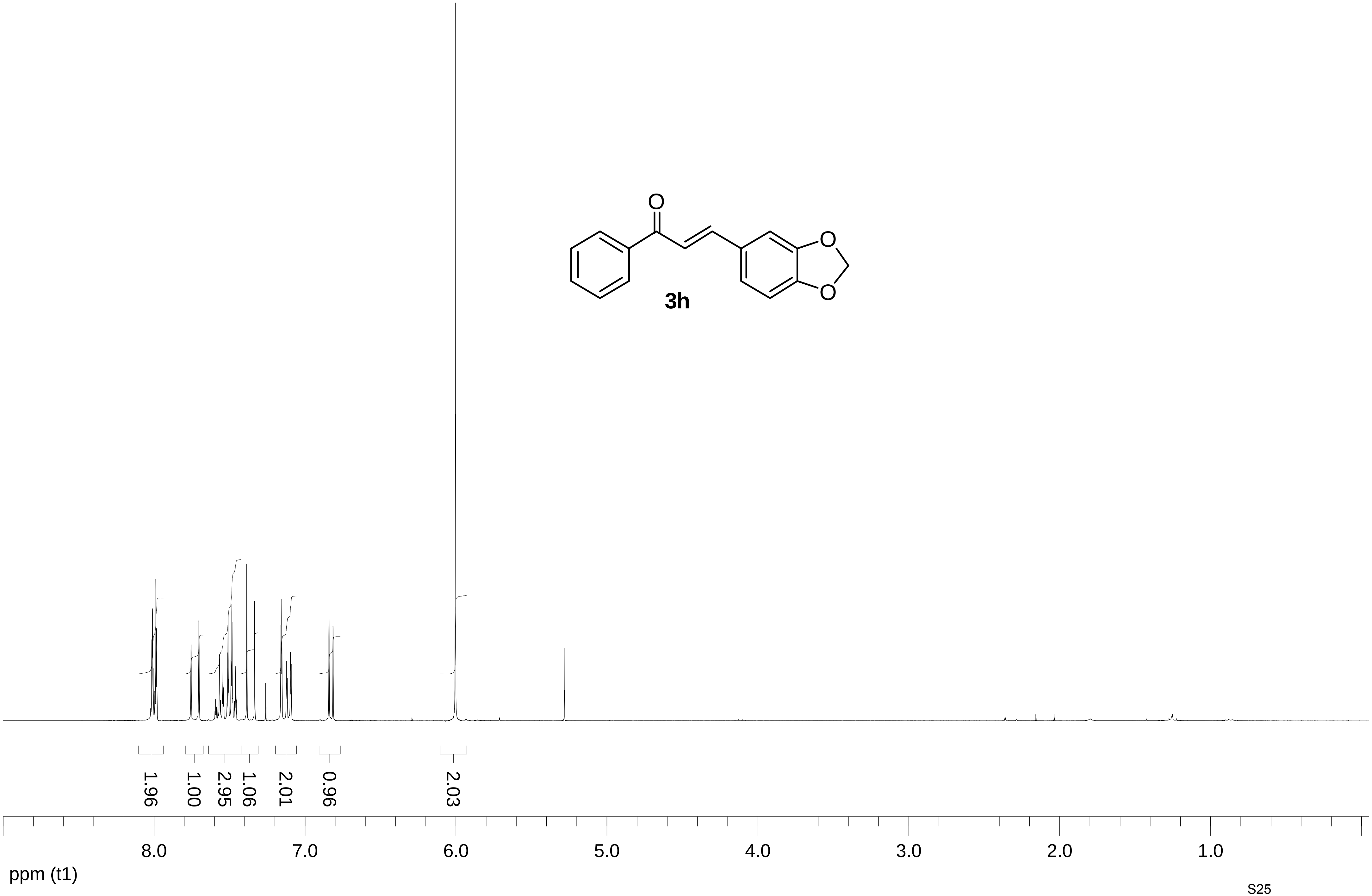

O
O
O
3h
1.96
1.00
2.95
1.06
2.01
0.96
2.03
8.0
7.0
6.0
5.0
4.0
3.0
2.0
1.0
ppm (t1)

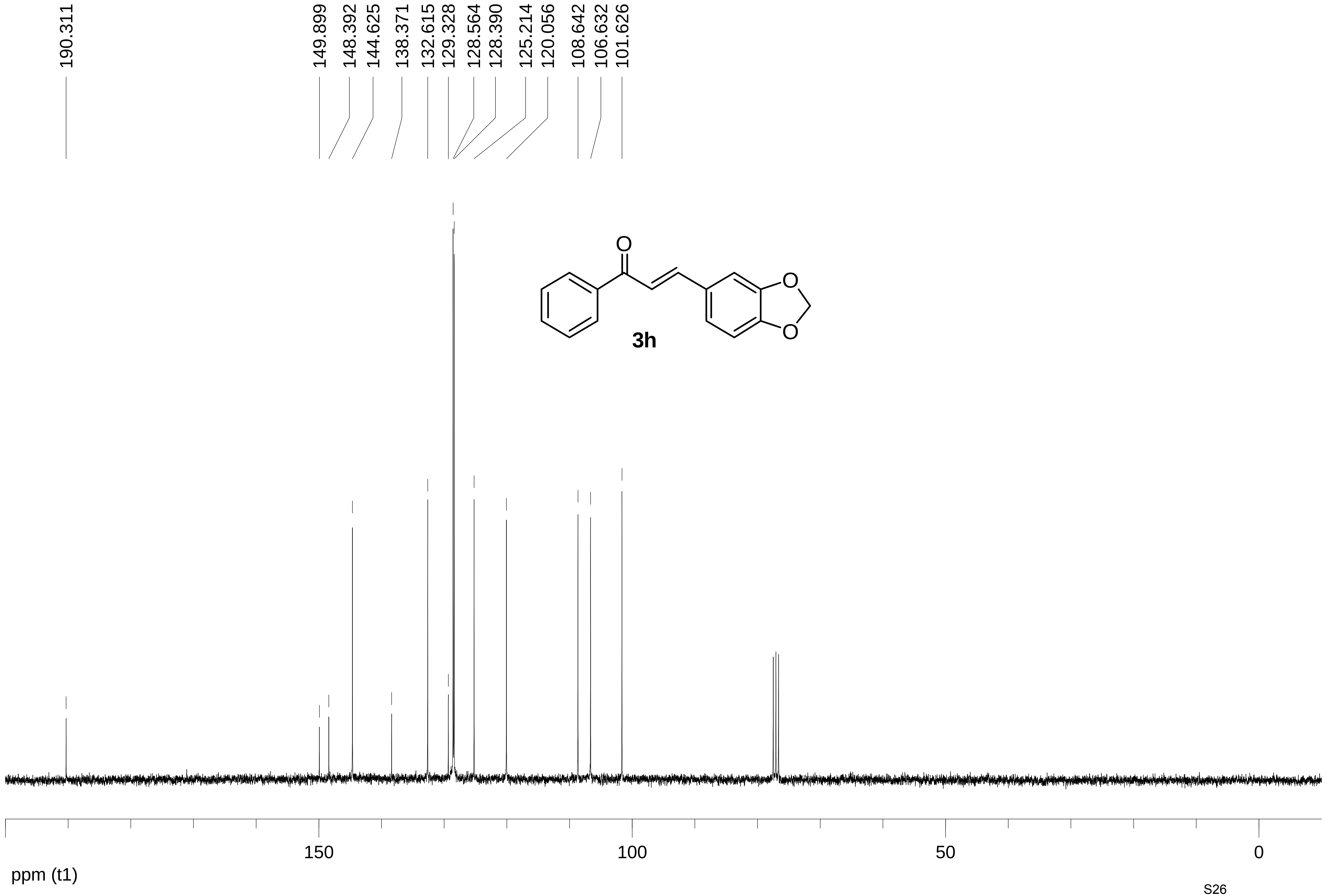
190.311
149.899
148.392
144.625
138.371
132.615
129.328
128.564
128.390
125.214
120.056
108.642
106.632
101.626
O
O
O
3h
150
100
50
0
ppm (t1)

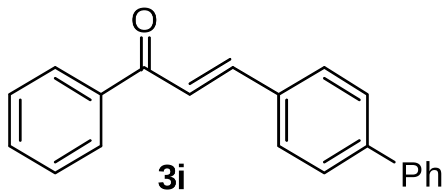

3i

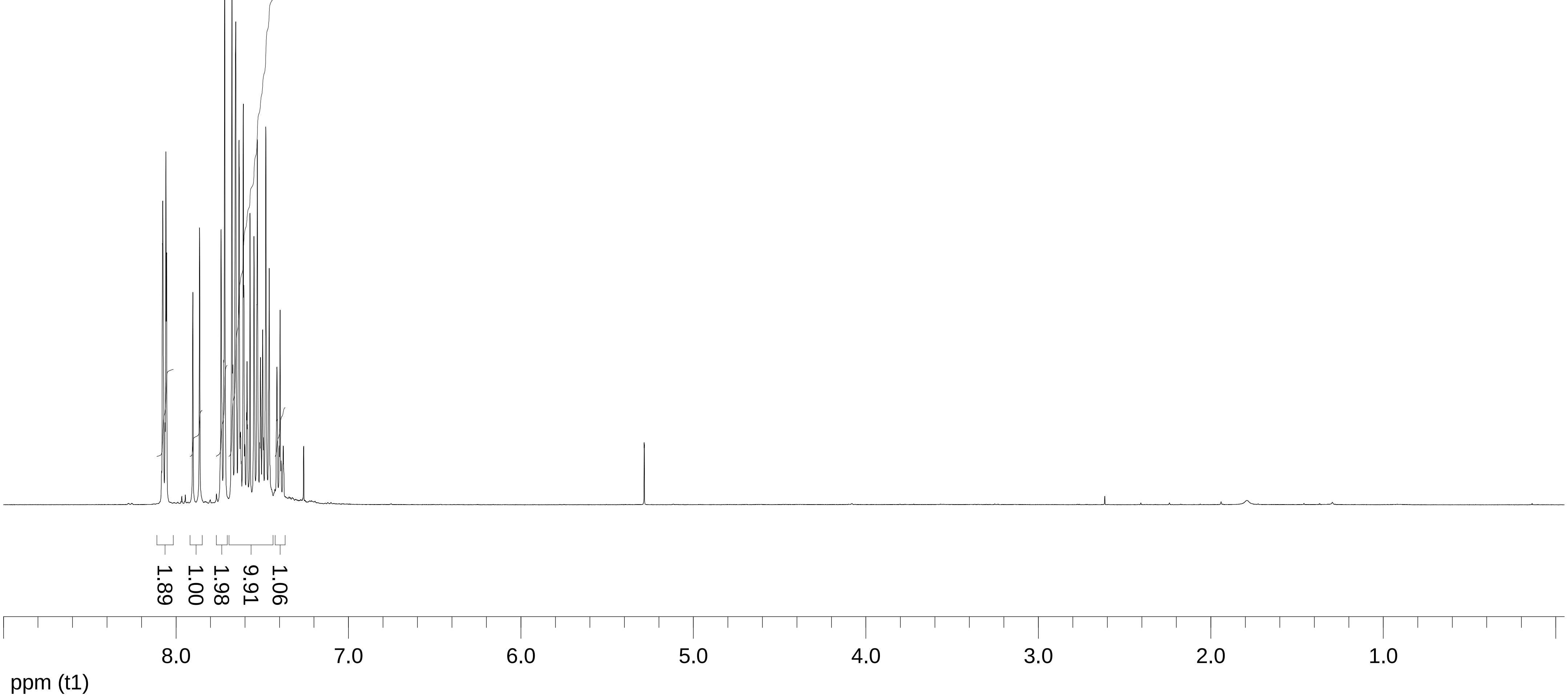

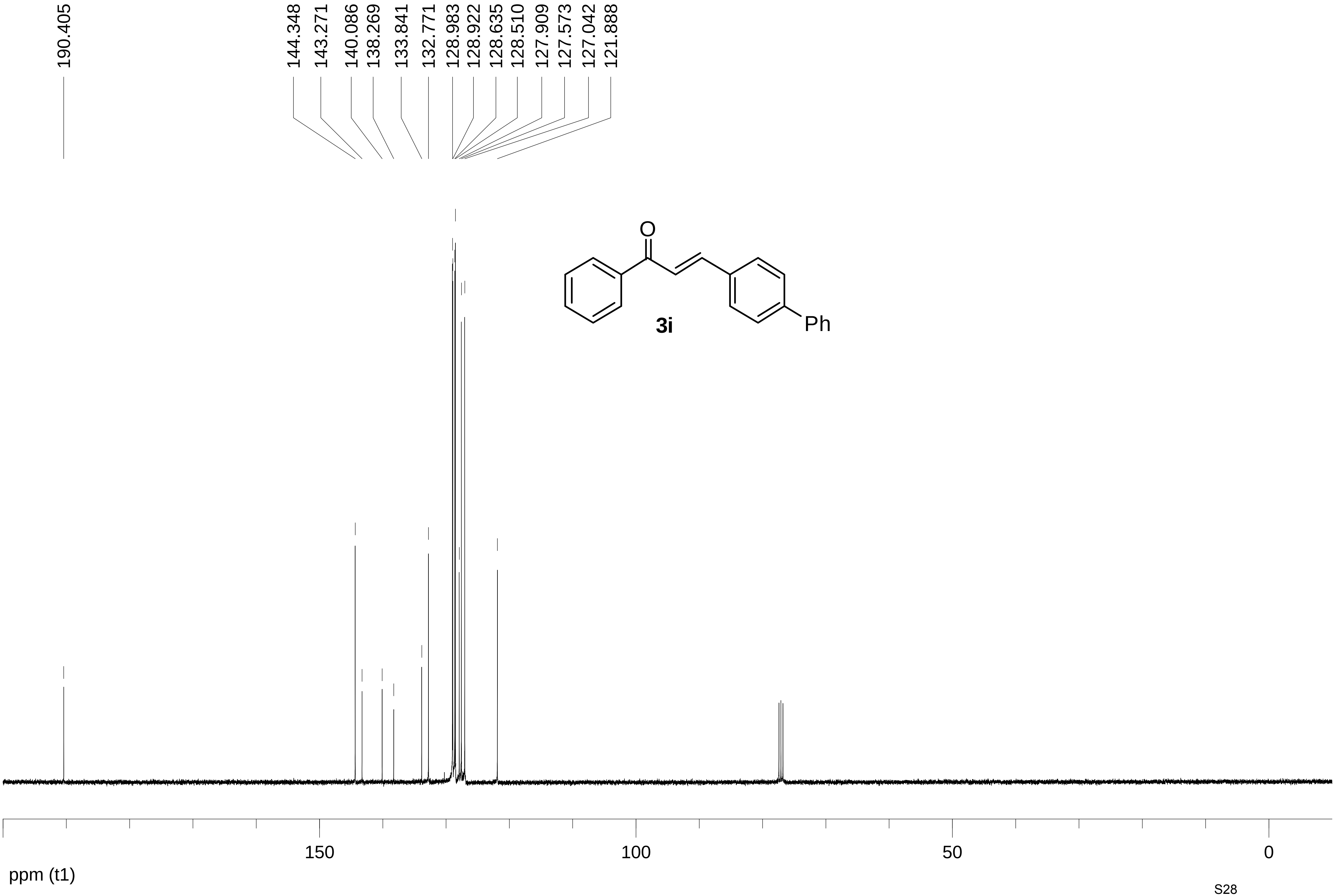
190.405
144.348
143.271
140.086
138.269
133.841
132.771
128.983
128.922
128.635
128.510
127.909
127.573
127.042
121.888
O
3i
Ph
150
100
50
0
ppm (t1)

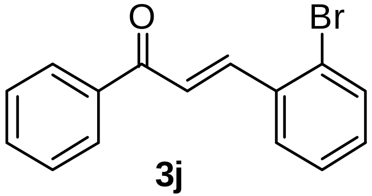

3j

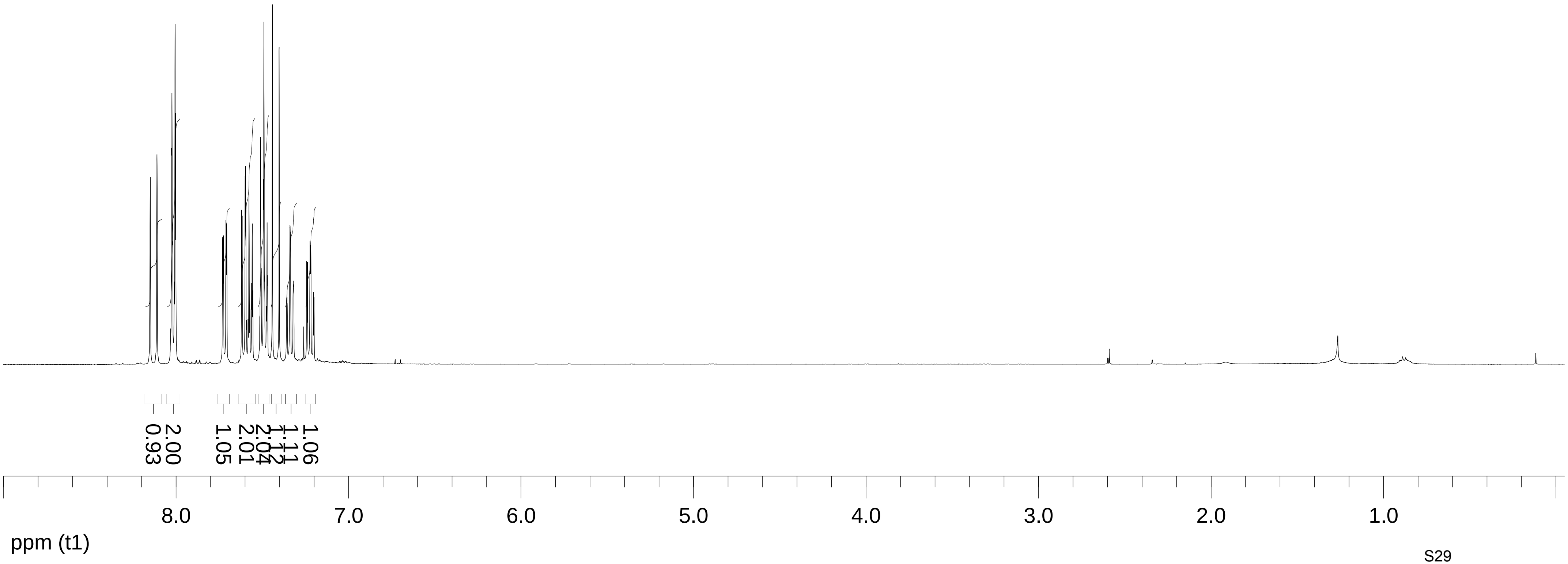

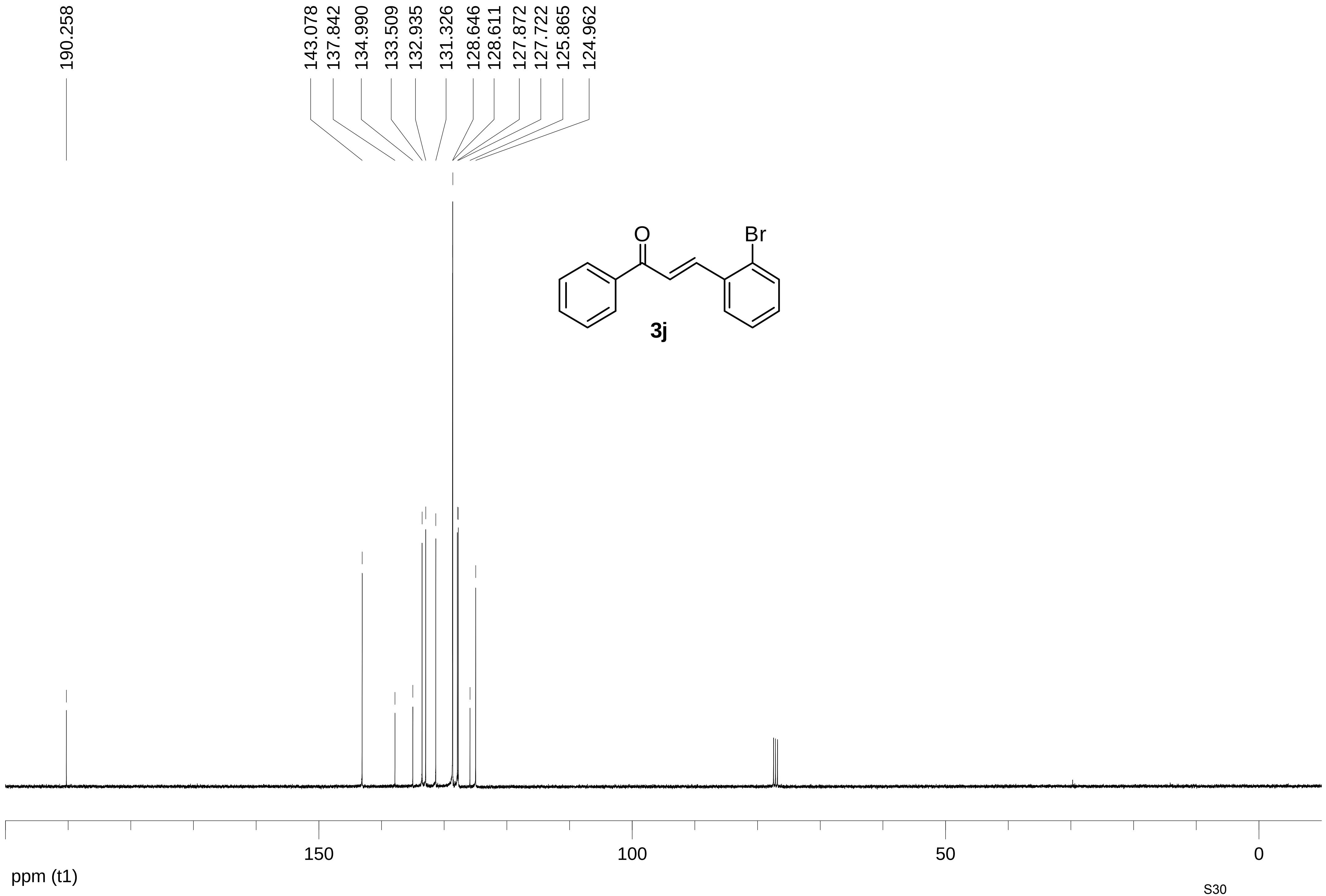
190.258
143.078
137.842
134.990
133.509
132.935
131.326
128.646
128.611
127.872
127.722
125.865
124.962
O
Br
3j
150
100
50
0
ppm (t1)

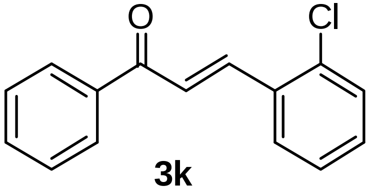

3k

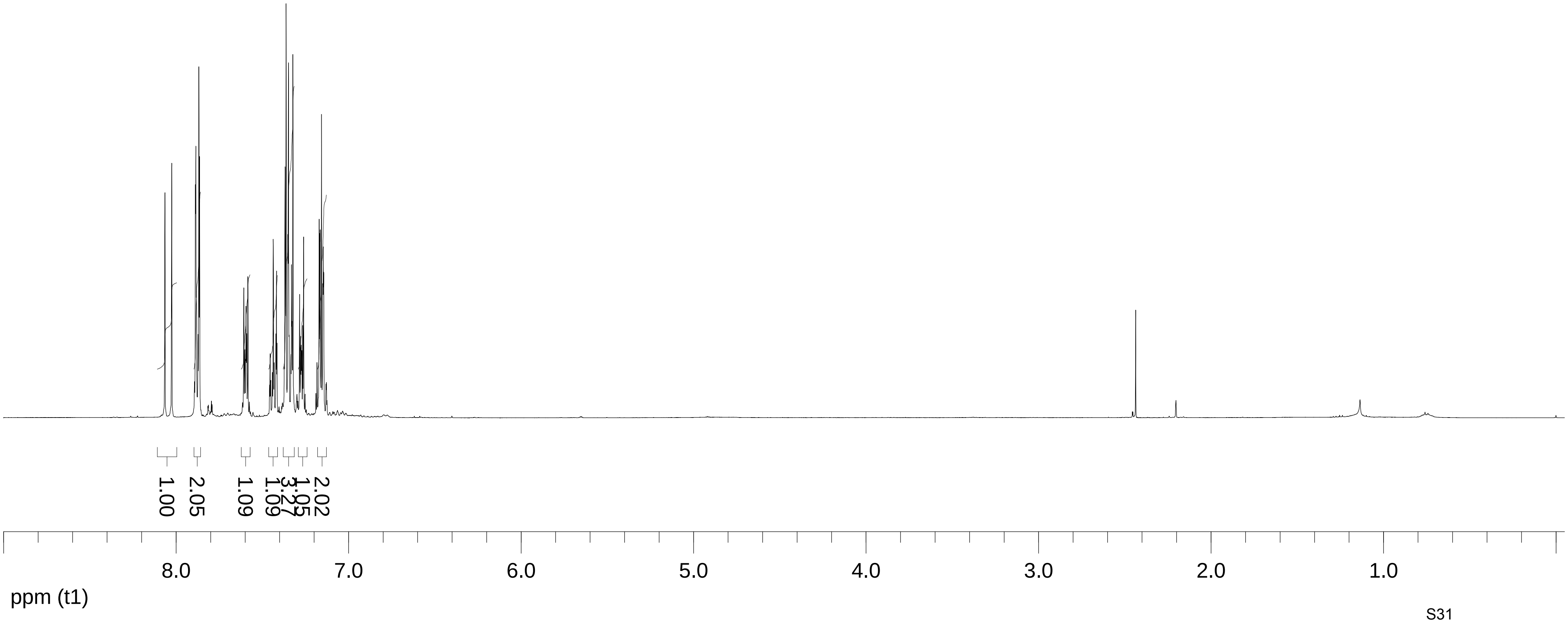

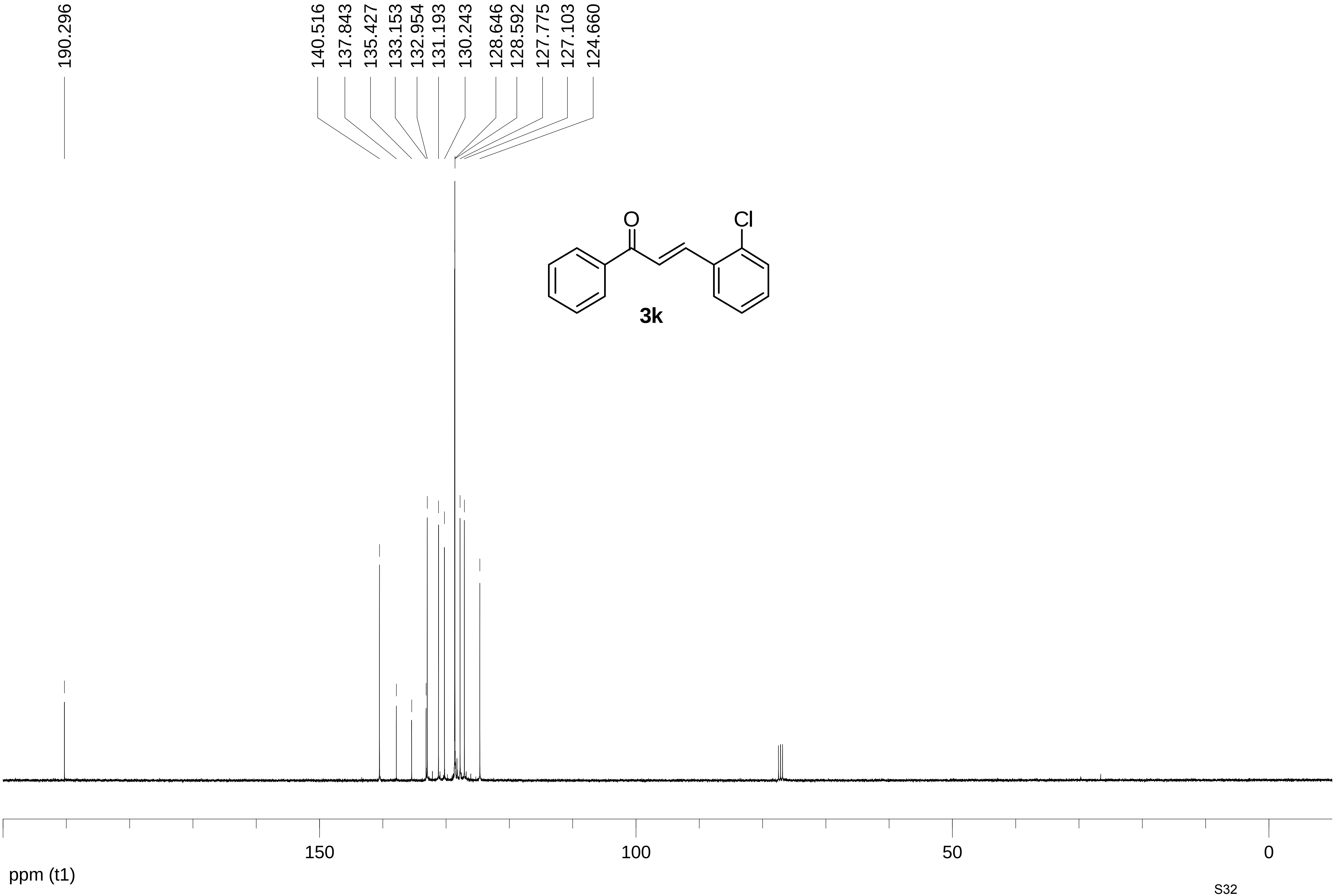
190.296
140.516
137.843
135.427
133.153
132.954
131.193
130.243
128.646
128.592
127.775
127.103
124.660
O
Cl
3k
150
100
50
0
ppm (t1)

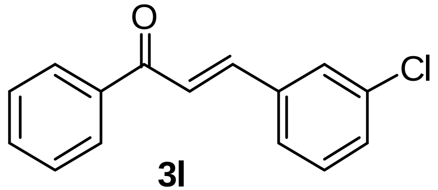

3l

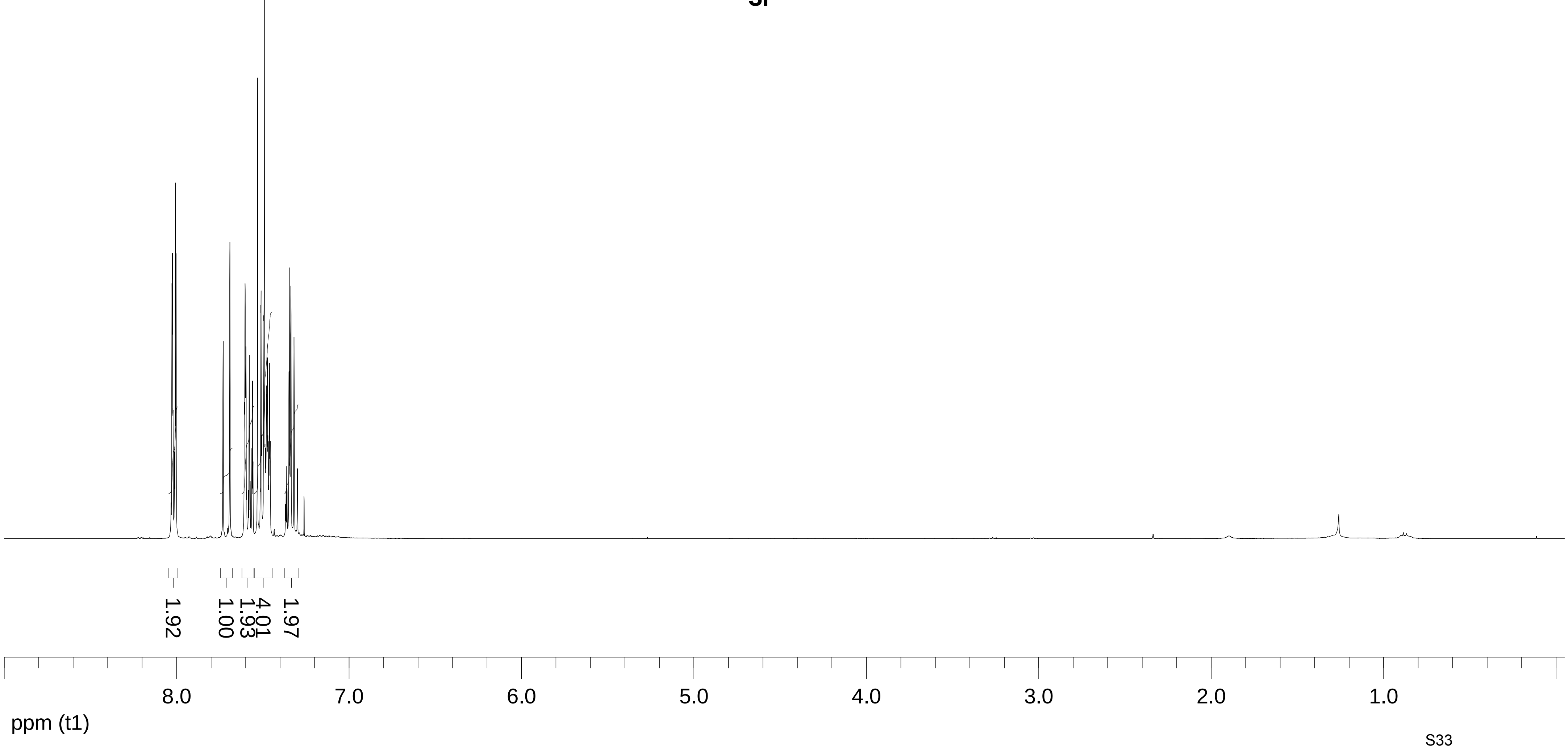

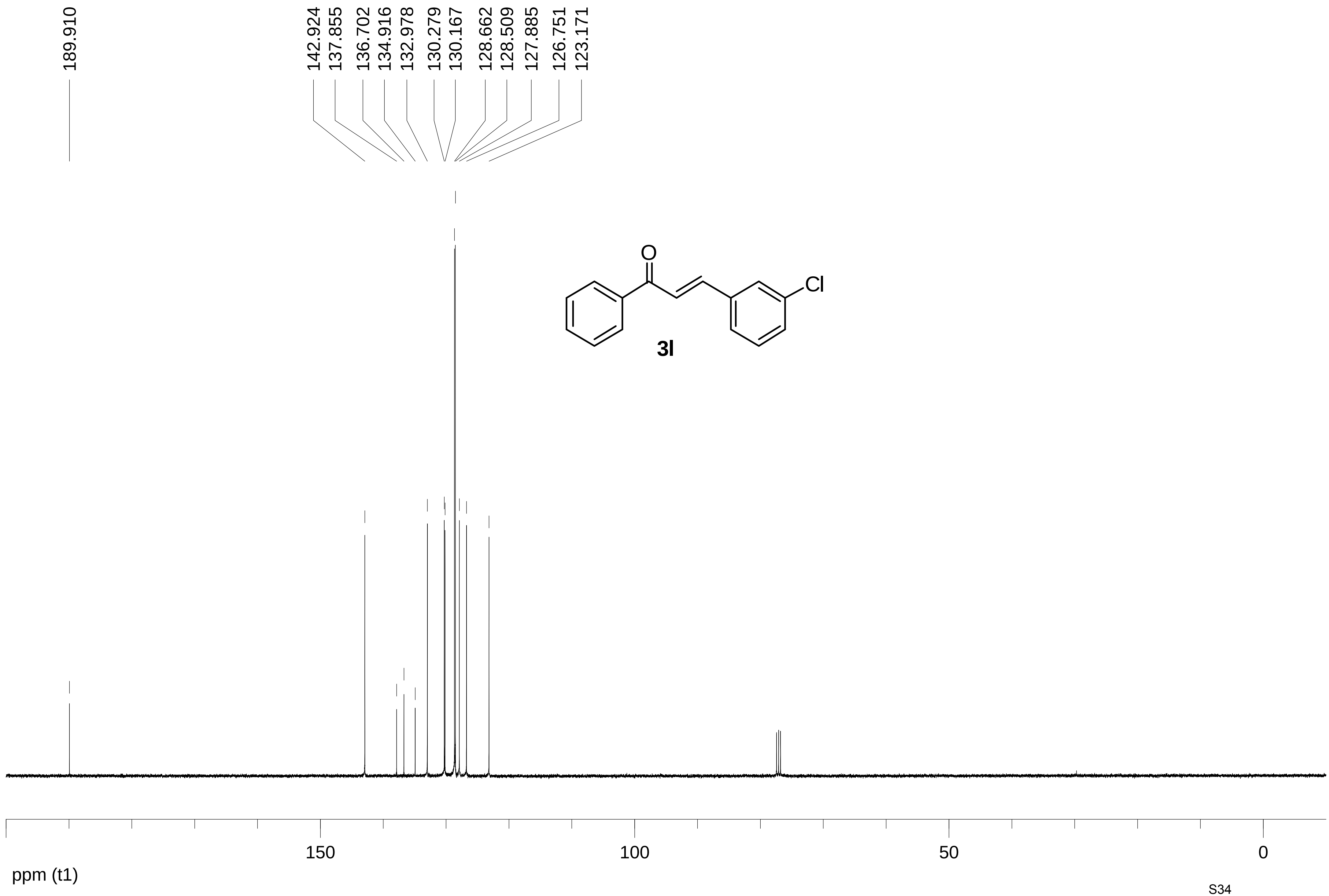
189.910
142.924
137.855
136.702
134.916
132.978
130.279
130.167
128.662
128.509
127.885
126.751
123.171
O
Cl
3l
150
100
50
0
ppm (t1)

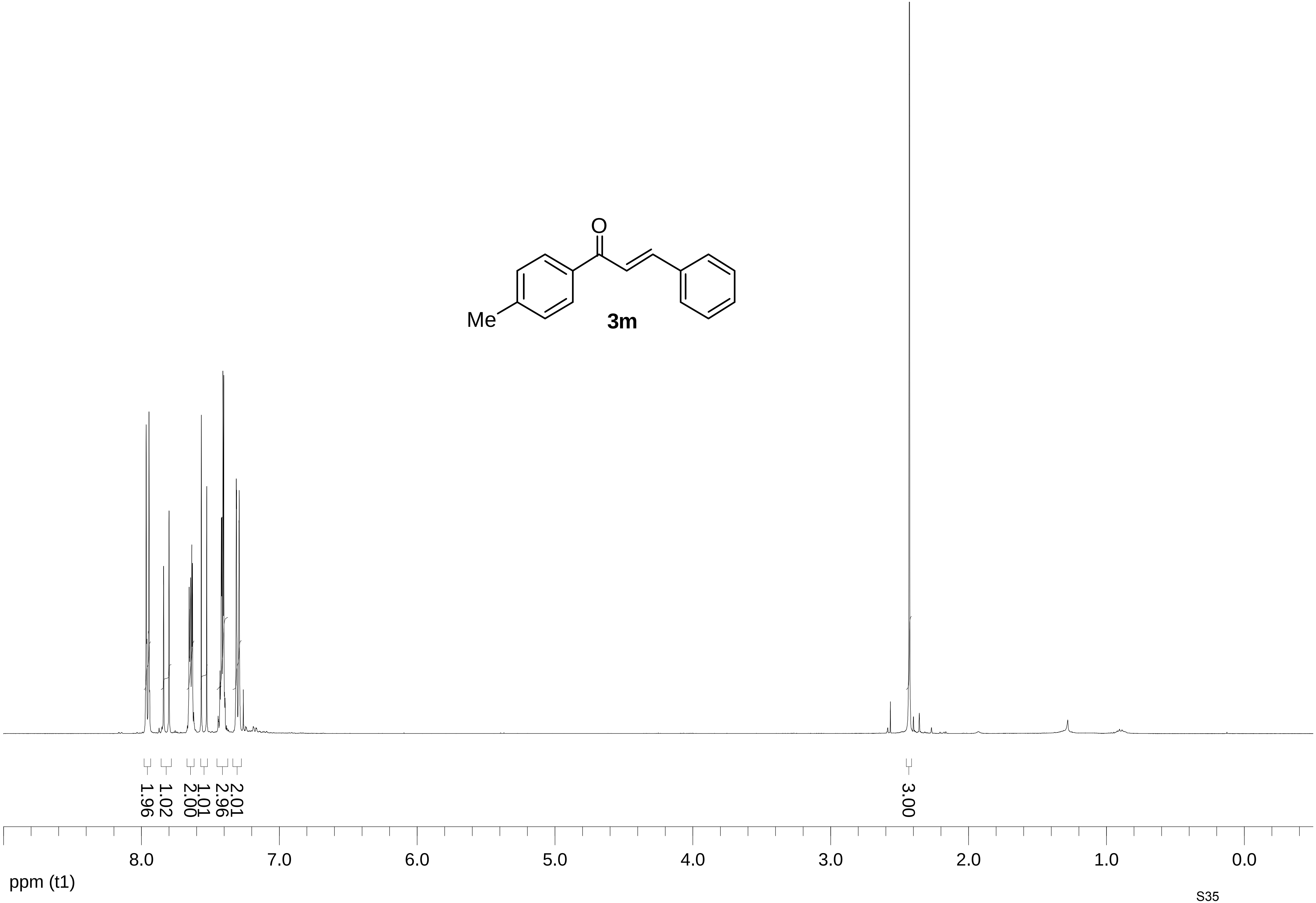
O
Me
3m
1.96
1.02
2.00
1.01
2.96
2.01
3.00
8.0
7.0
6.0
5.0
4.0
3.0
2.0
1.0
0.0
ppm (t1)

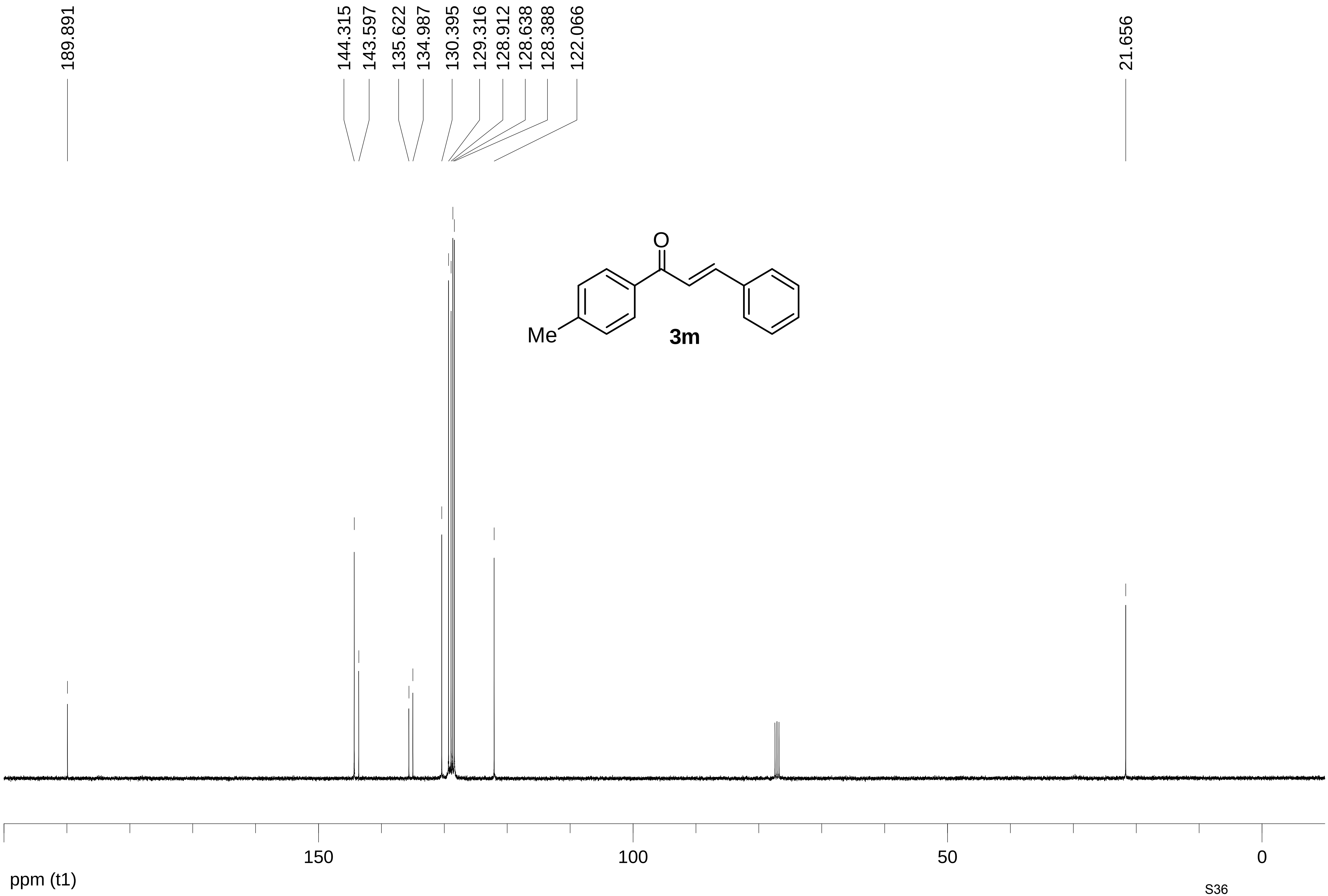
189.891
144.315
143.597
135.622
134.987
130.395
129.316
128.912
128.638
128.388
122.066
21.656
O
Me
3m
150
100
50
0
ppm (t1)

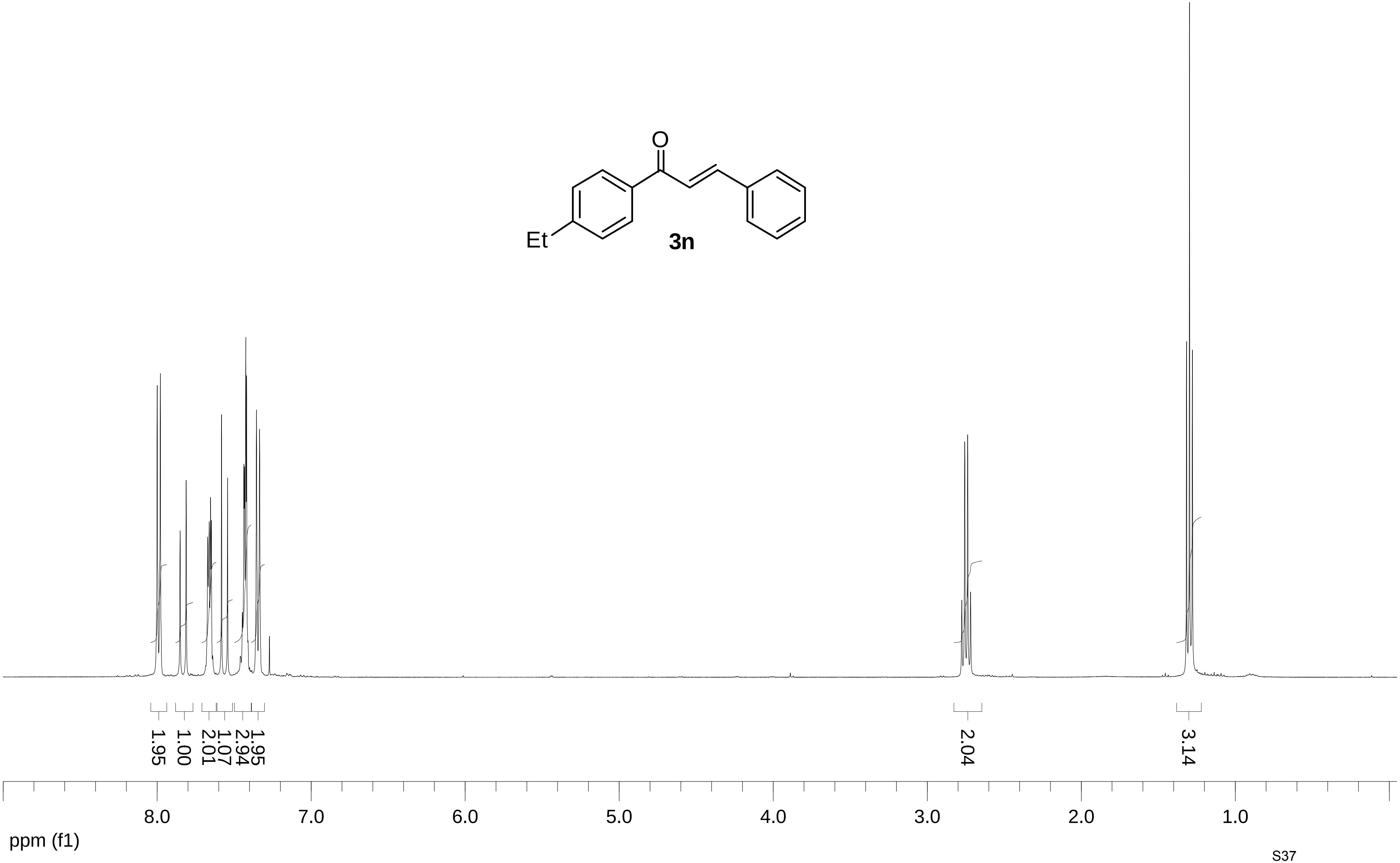
O
Et
3n
1.95
1.00
2.01
1.07
2.94
1.95
2.04
3.14
8.0
7.0
6.0
5.0
4.0
3.0
2.0
1.0
ppm (f1)

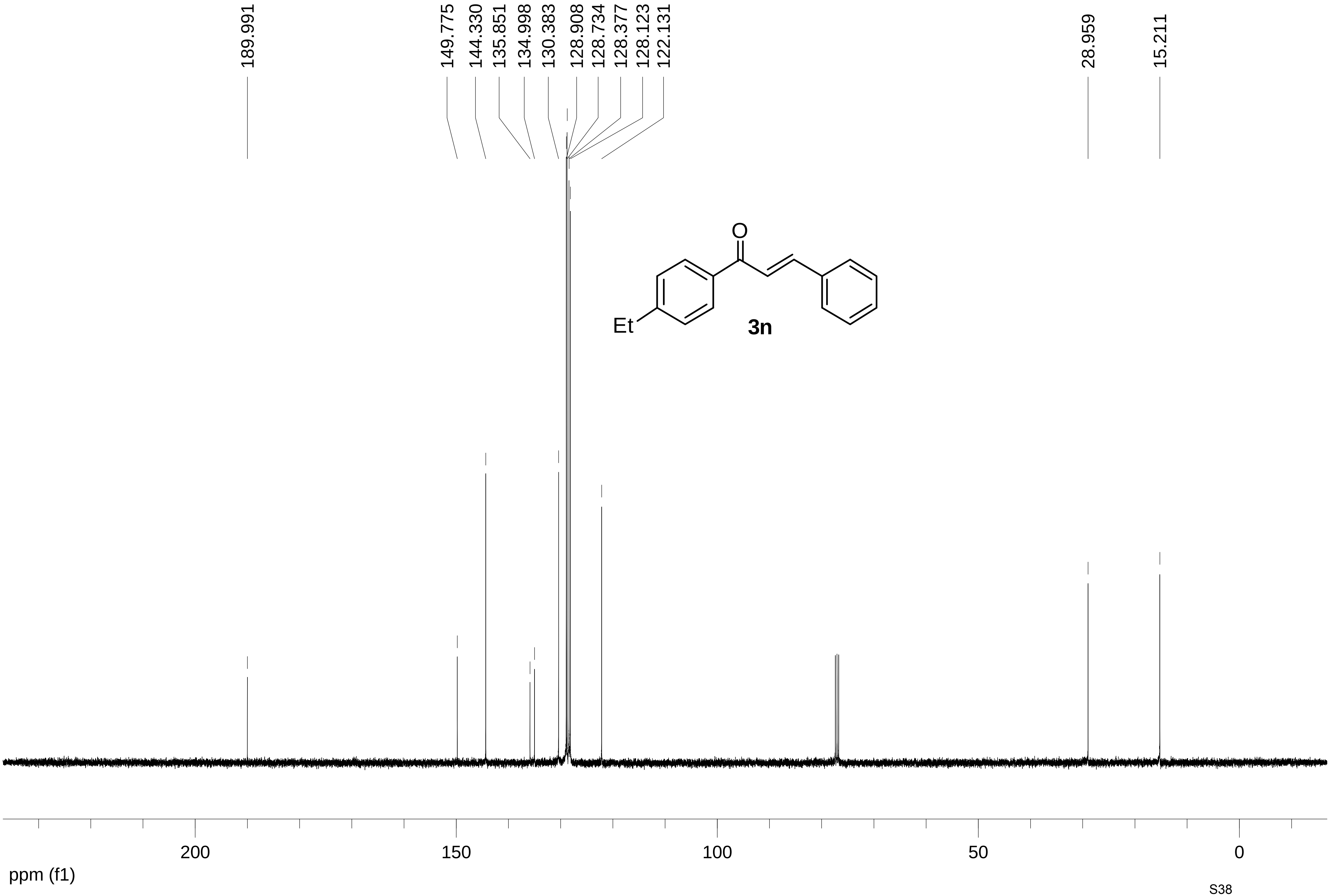
189.991
149.775
144.330
135.851
134.998
130.383
128.908
128.734
128.377
128.123
122.131
28.959
15.211
O
Et
3n
200
150
100
50
0
ppm (f1)

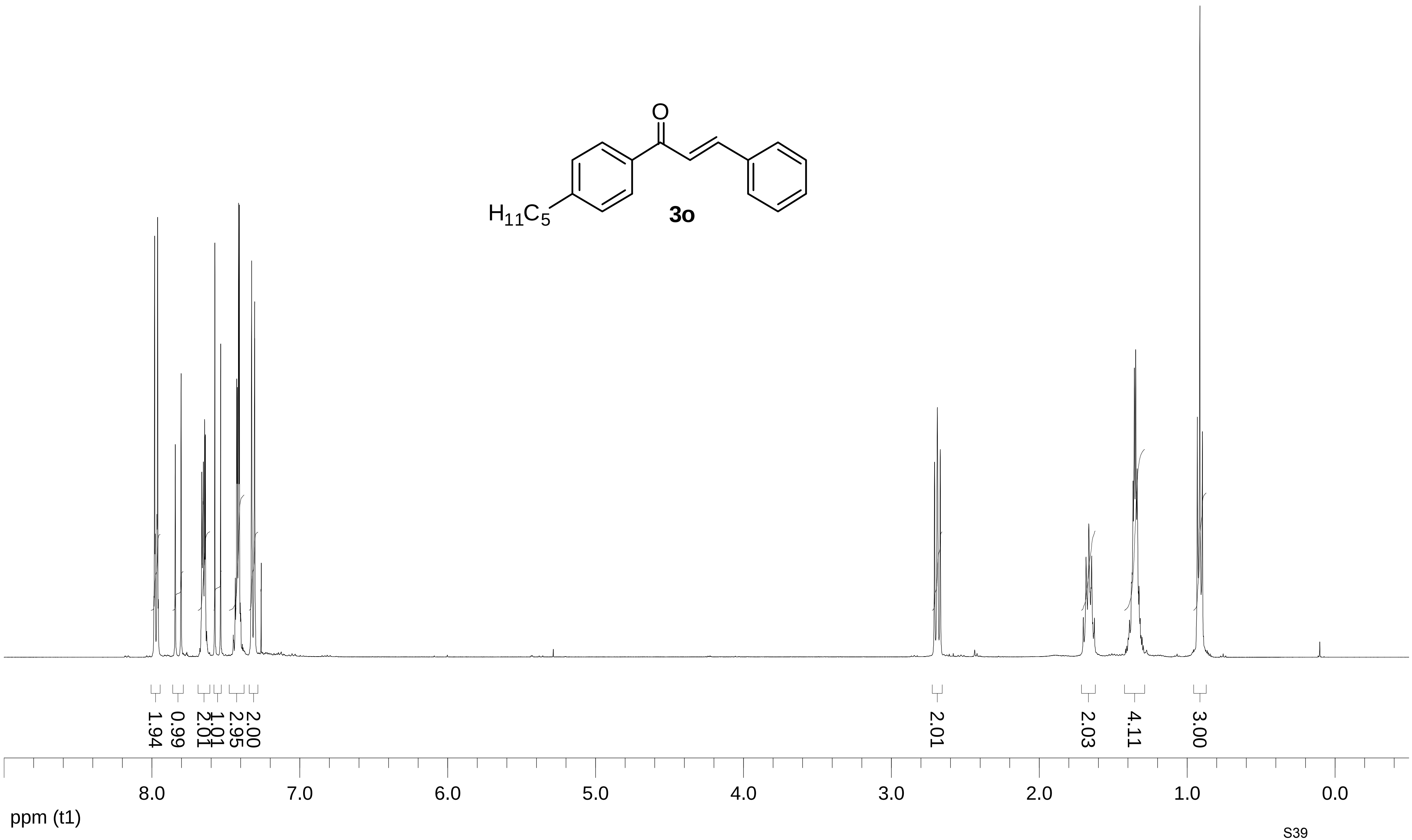
O
H11C5
3o
1.94
0.99
2.01
1.01
2.95
2.00
2.01
2.03
4.11
3.00
8.0
7.0
6.0
5.0
4.0
3.0
2.0
1.0
0.0
ppm (t1)

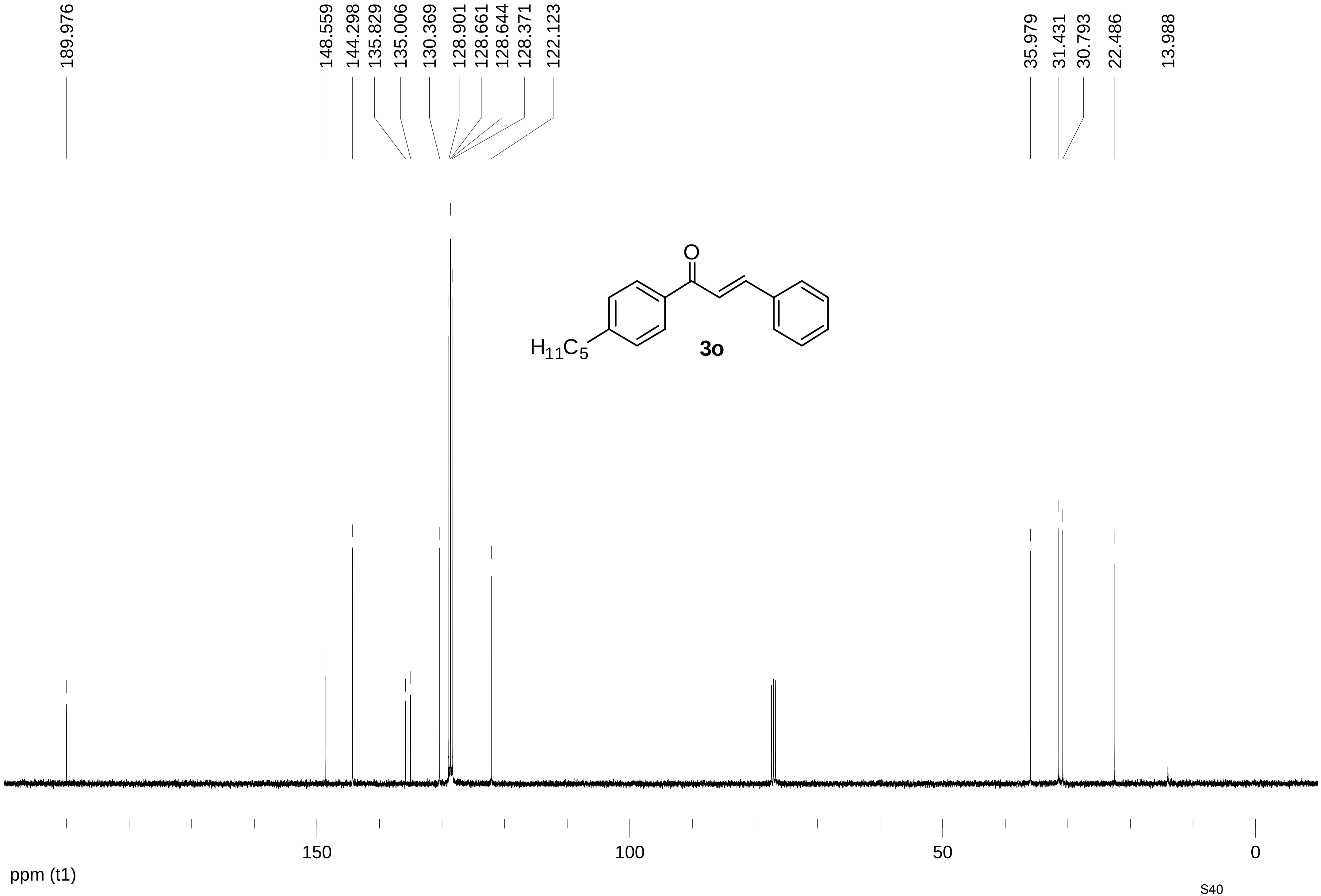
189.976
148.559
144.298
135.829
135.006
130.369
128.901
128.661
128.644
128.371
122.123
35.979
31.431
30.793
22.486
13.988
O
$H_{11}C_5$
3o
150
100
50
0
ppm (t1)

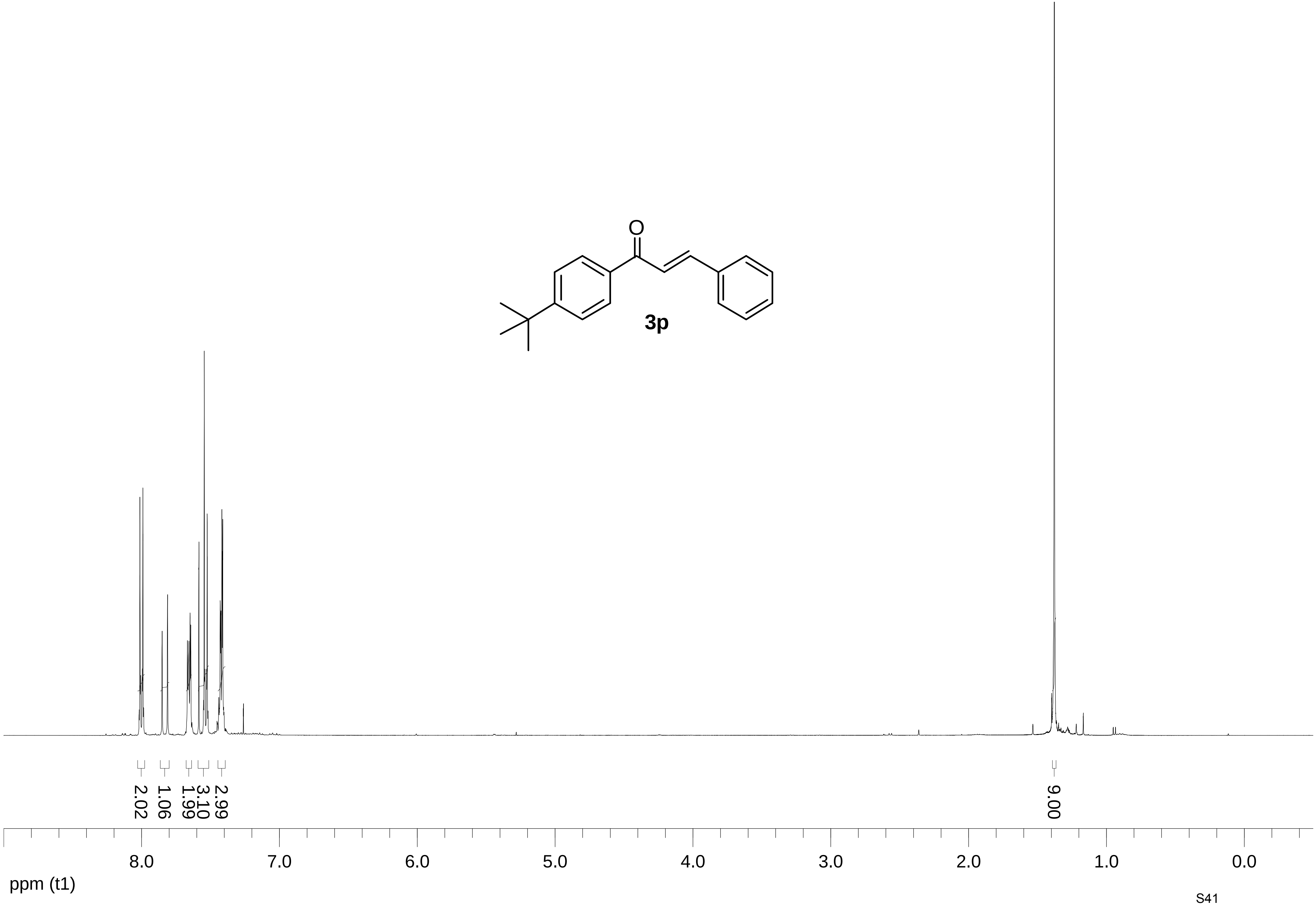
O
3p
2.02
1.06
1.99
3.10
2.99
9.00
8.0
7.0
6.0
5.0
4.0
3.0
2.0
1.0
0.0
ppm (t1)

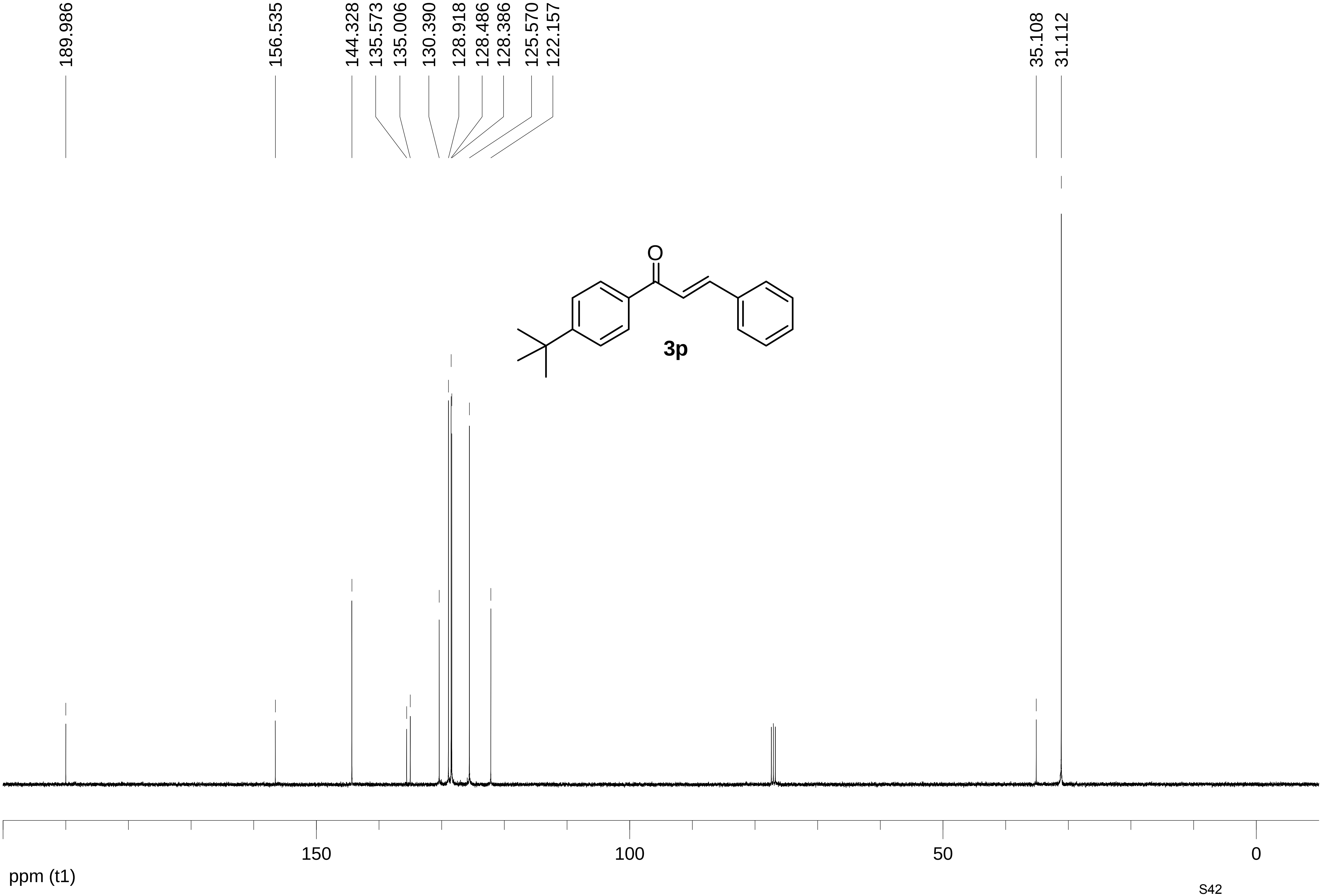
189.986
156.535
144.328
135.573
135.006
130.390
128.918
128.486
128.386
125.570
122.157
35.108
31.112
O
3p
150
100
50
0
ppm (t1)

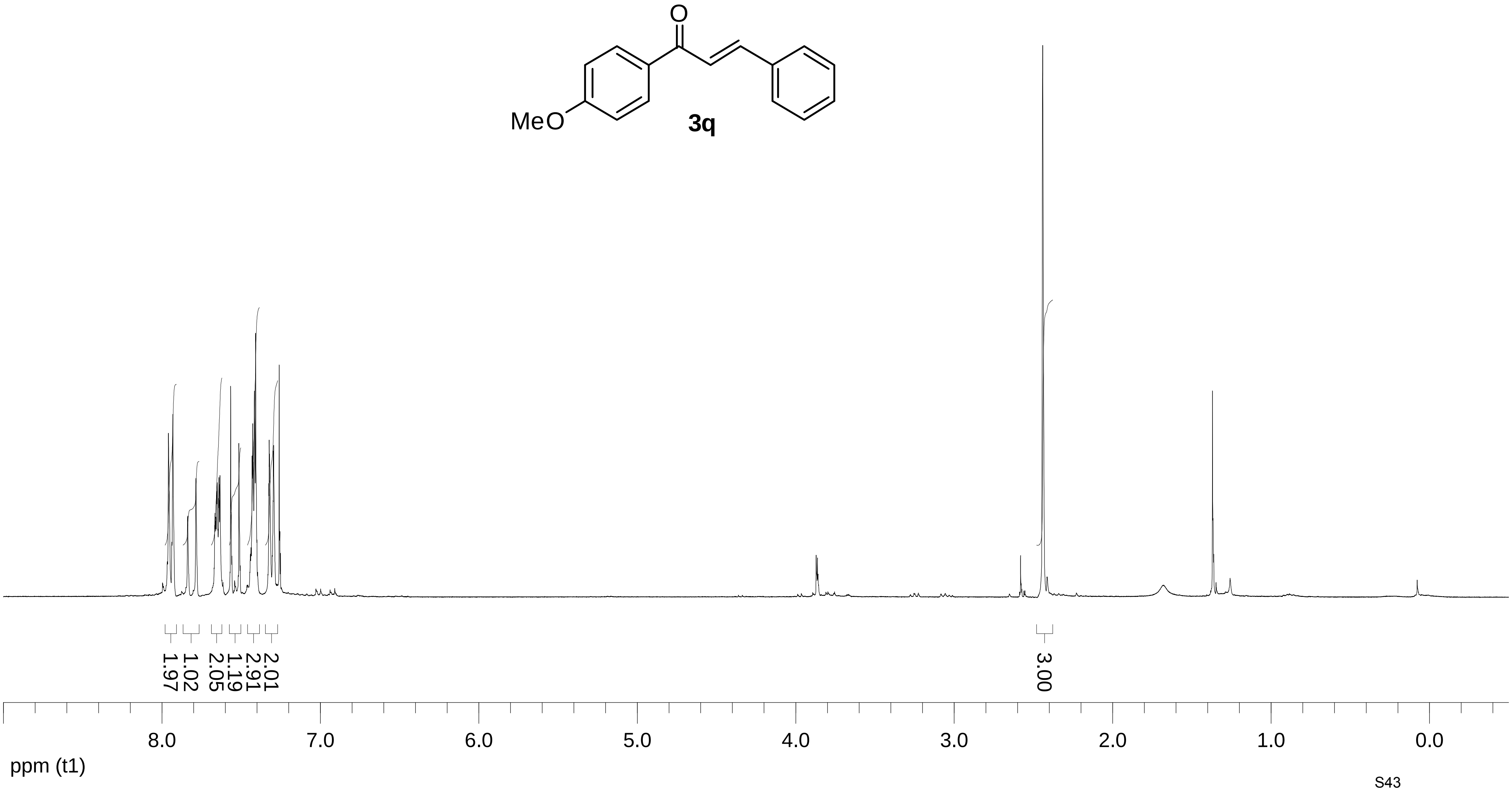
O
MeO
3q
1.97
1.02
2.05
1.19
2.91
2.01
3.00
8.0
7.0
6.0
5.0
4.0
3.0
2.0
1.0
0.0
ppm (t1)

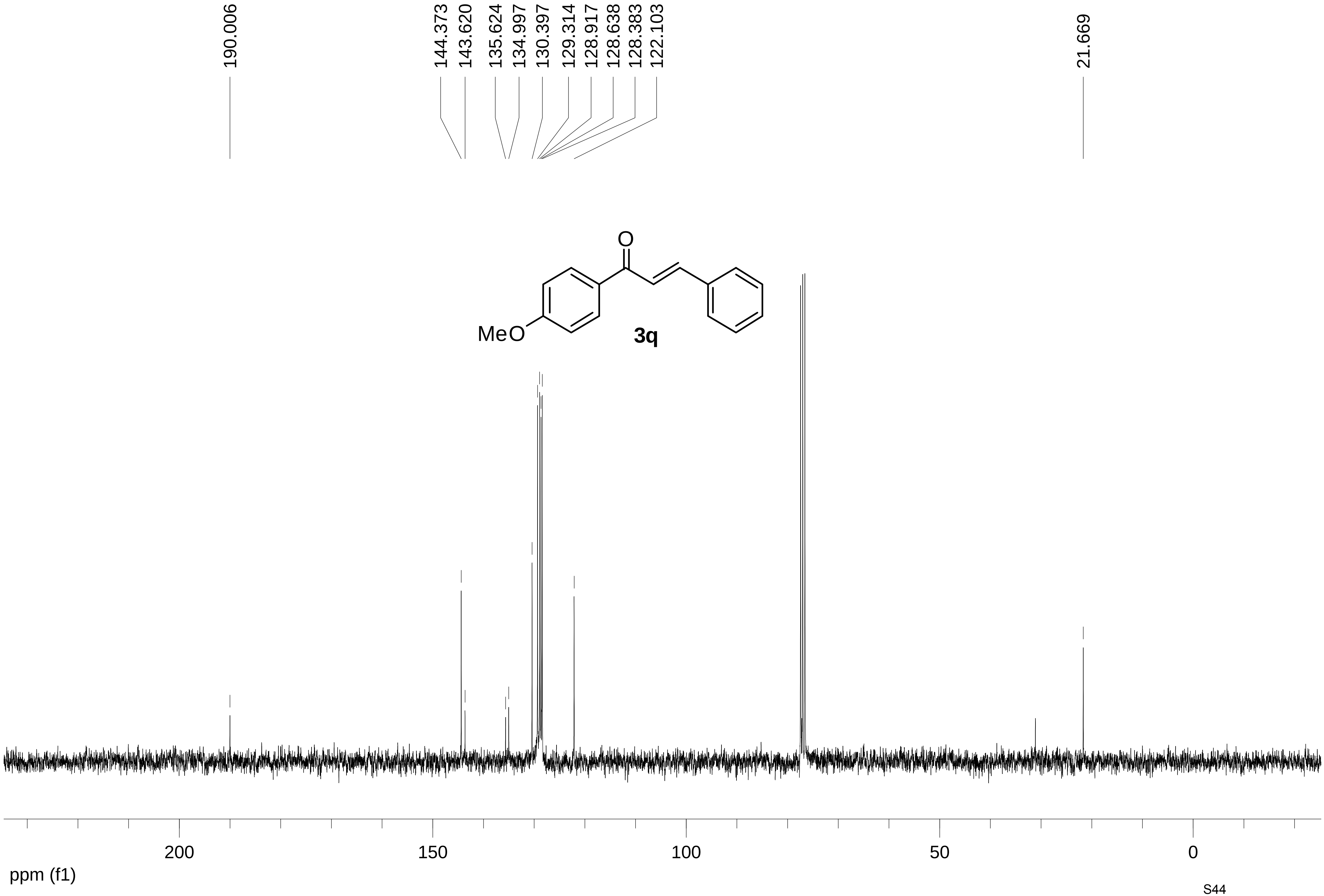
190.006
144.373
143.620
135.624
134.997
130.397
129.314
128.917
128.638
128.383
122.103
21.669
O
MeO
3q
200
150
100
50
0
ppm (f1)

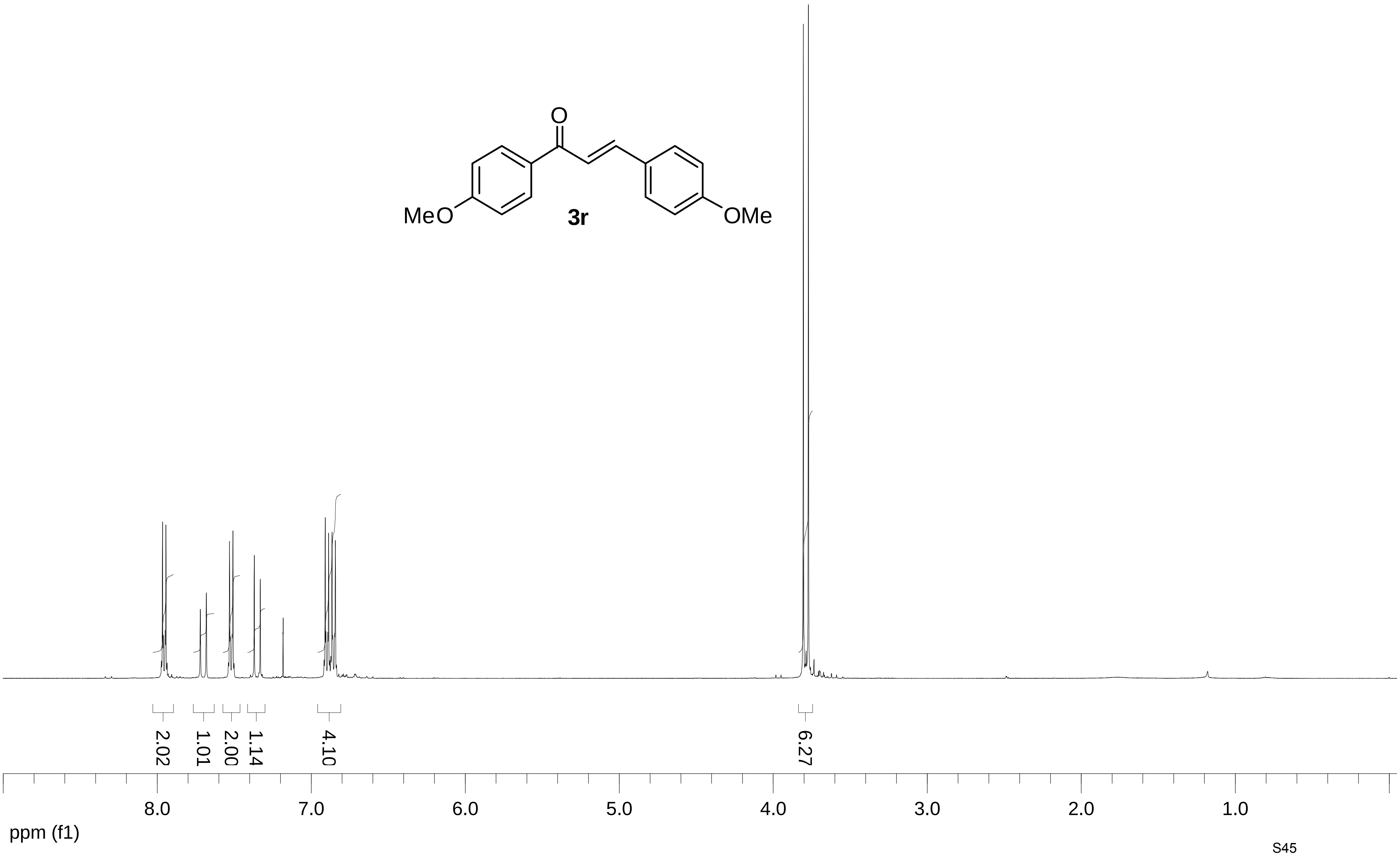

O
MeO
3r
OMe
2.02
1.01
2.00
1.14
4.10
6.27
8.0
7.0
6.0
5.0
4.0
3.0
2.0
1.0
ppm (f1)

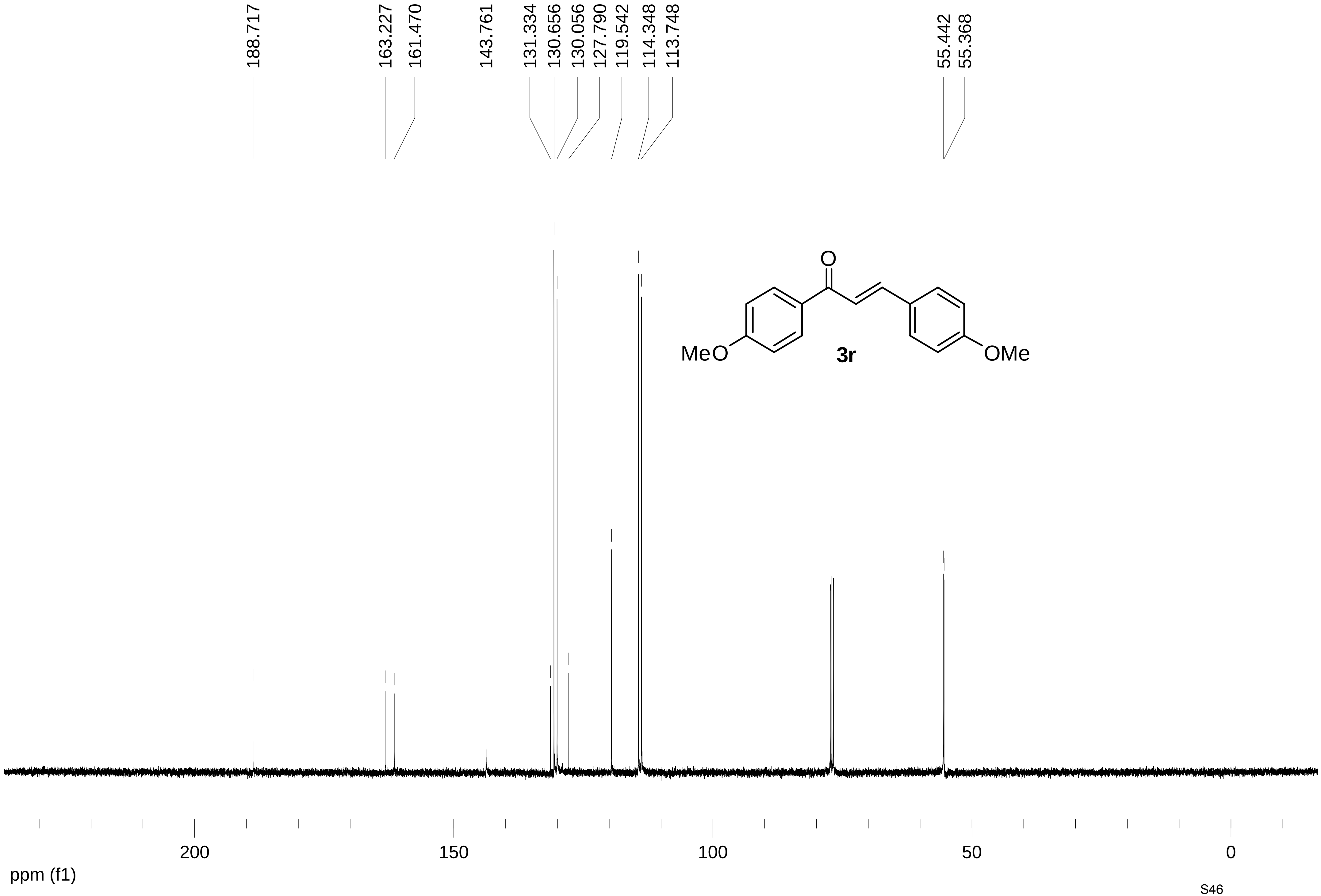
188.717
163.227
161.470
143.761
131.334
130.656
130.056
127.790
119.542
114.348
113.748
55.442
55.368
O
MeO
3r
OMe
200
150
100
50
0
ppm (f1)

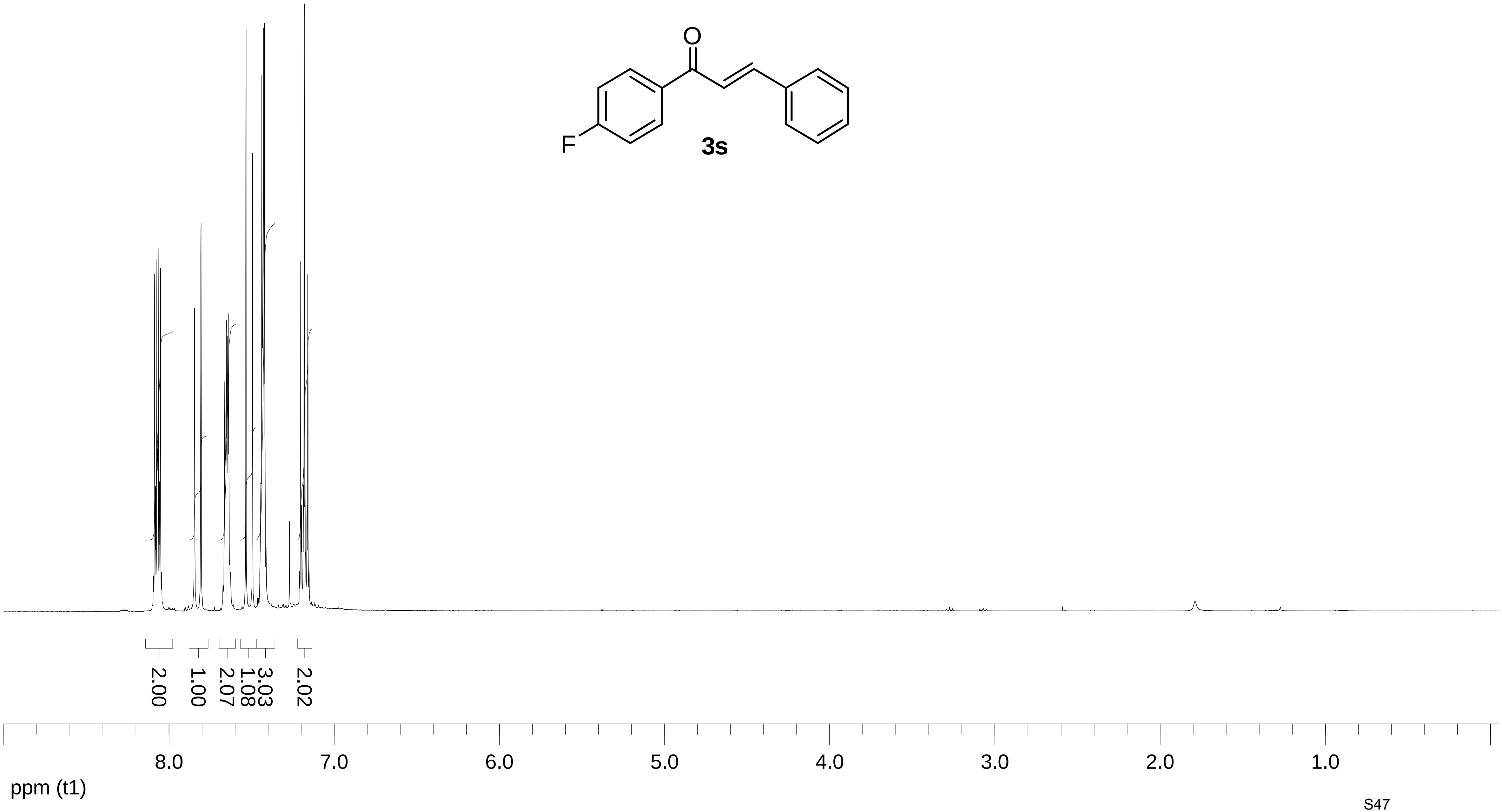

O
F
3s
2.00
1.00
2.07
1.08
3.03
2.02
8.0
7.0
6.0
5.0
4.0
3.0
2.0
1.0
ppm (t1)

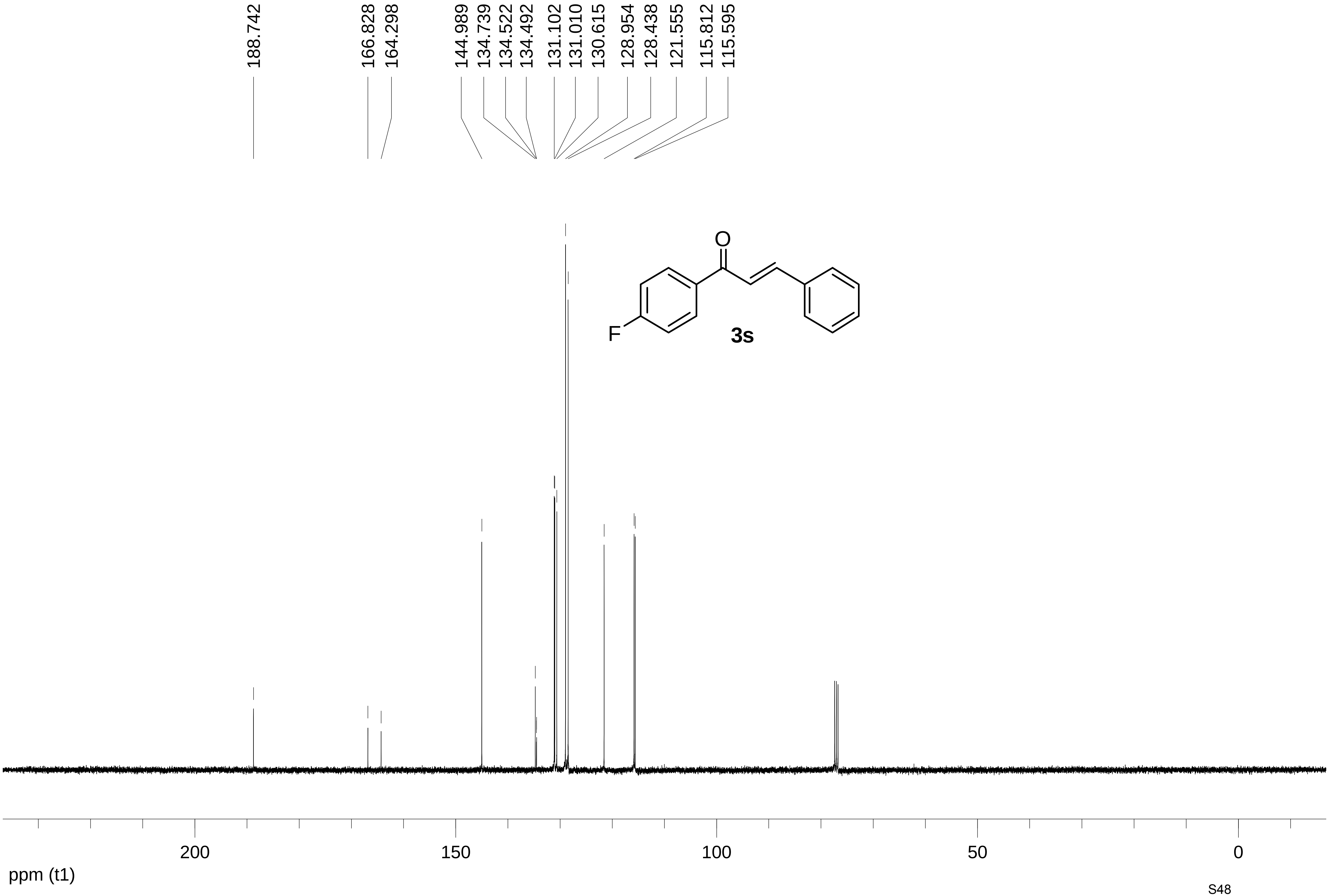
188.742
166.828
164.298
144.989
134.739
134.522
134.492
131.102
131.010
130.615
128.954
128.438
121.555
115.812
115.595
O
F
3s
200
150
100
50
0
ppm (t1)

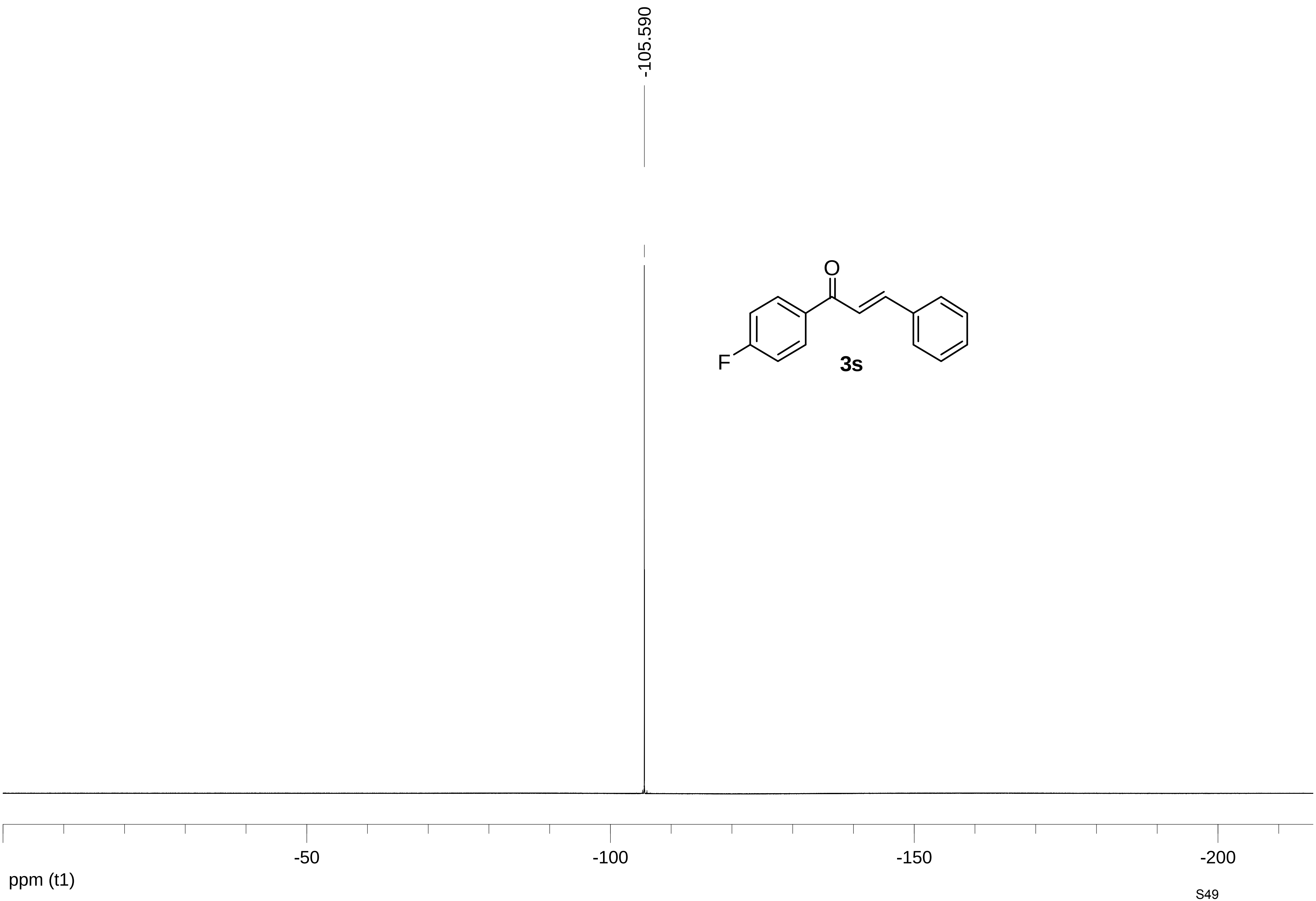

-105.590
O
F
3s
-50
-100
-150
-200
ppm (t1)

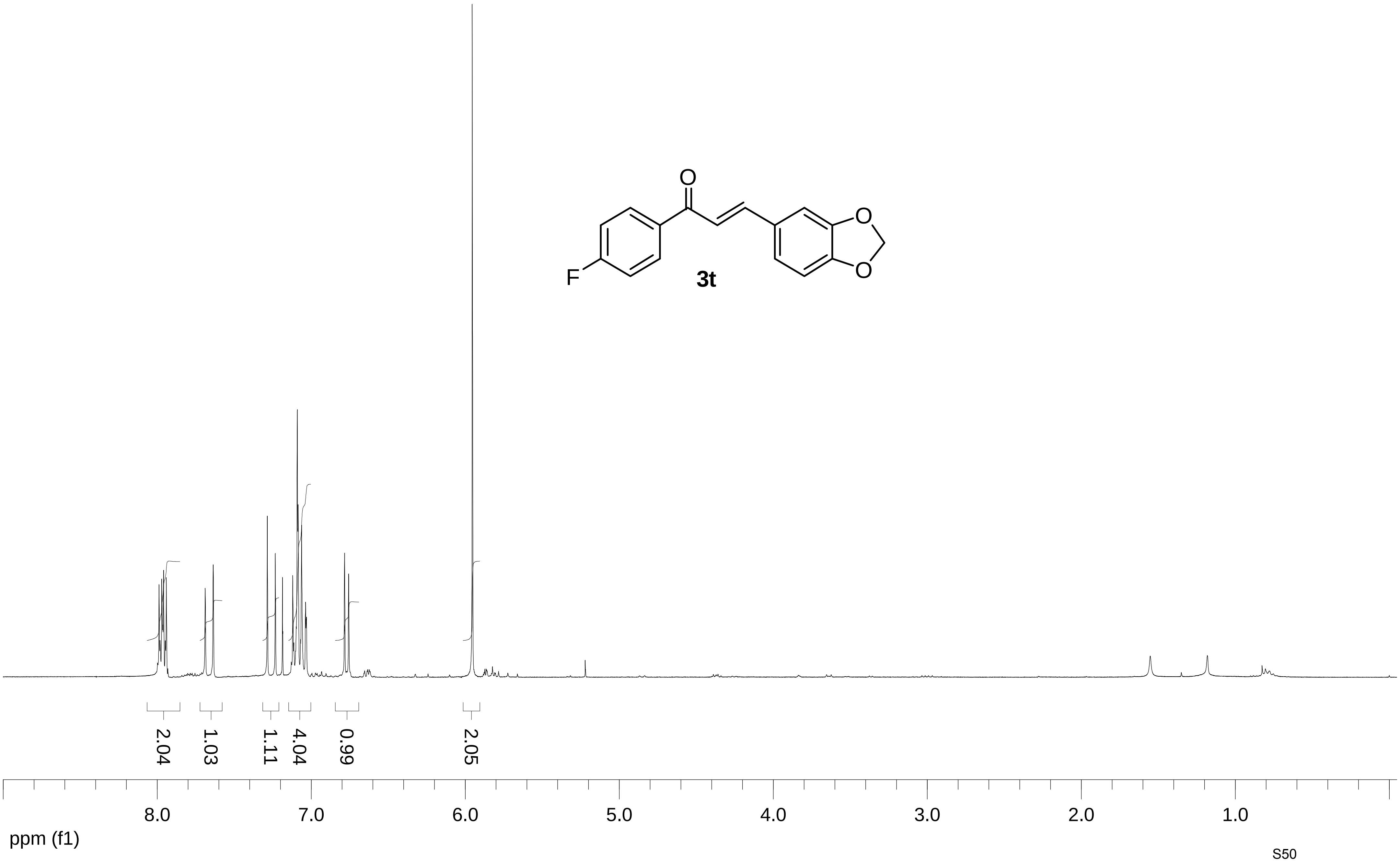

F
O
O
O
3t
2.04
1.03
1.11
4.04
0.99
2.05
8.0
7.0
6.0
5.0
4.0
3.0
2.0
1.0
ppm (f1)

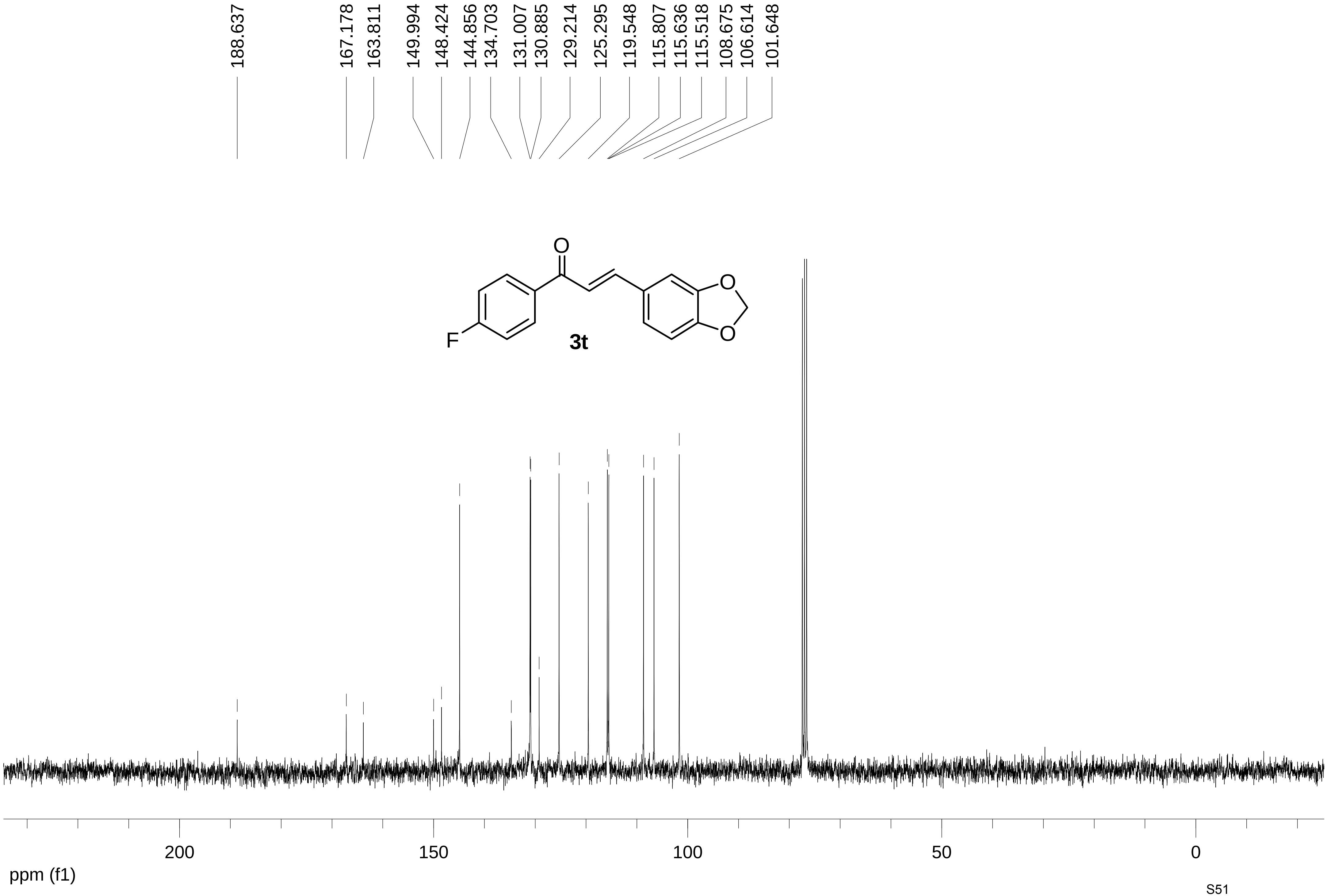
188.637
167.178
163.811
149.994
148.424
144.856
134.703
131.007
130.885
129.214
125.295
119.548
115.807
115.636
115.518
108.675
106.614
101.648
O
F
3t
O
O
200
150
100
50
0
ppm (f1)

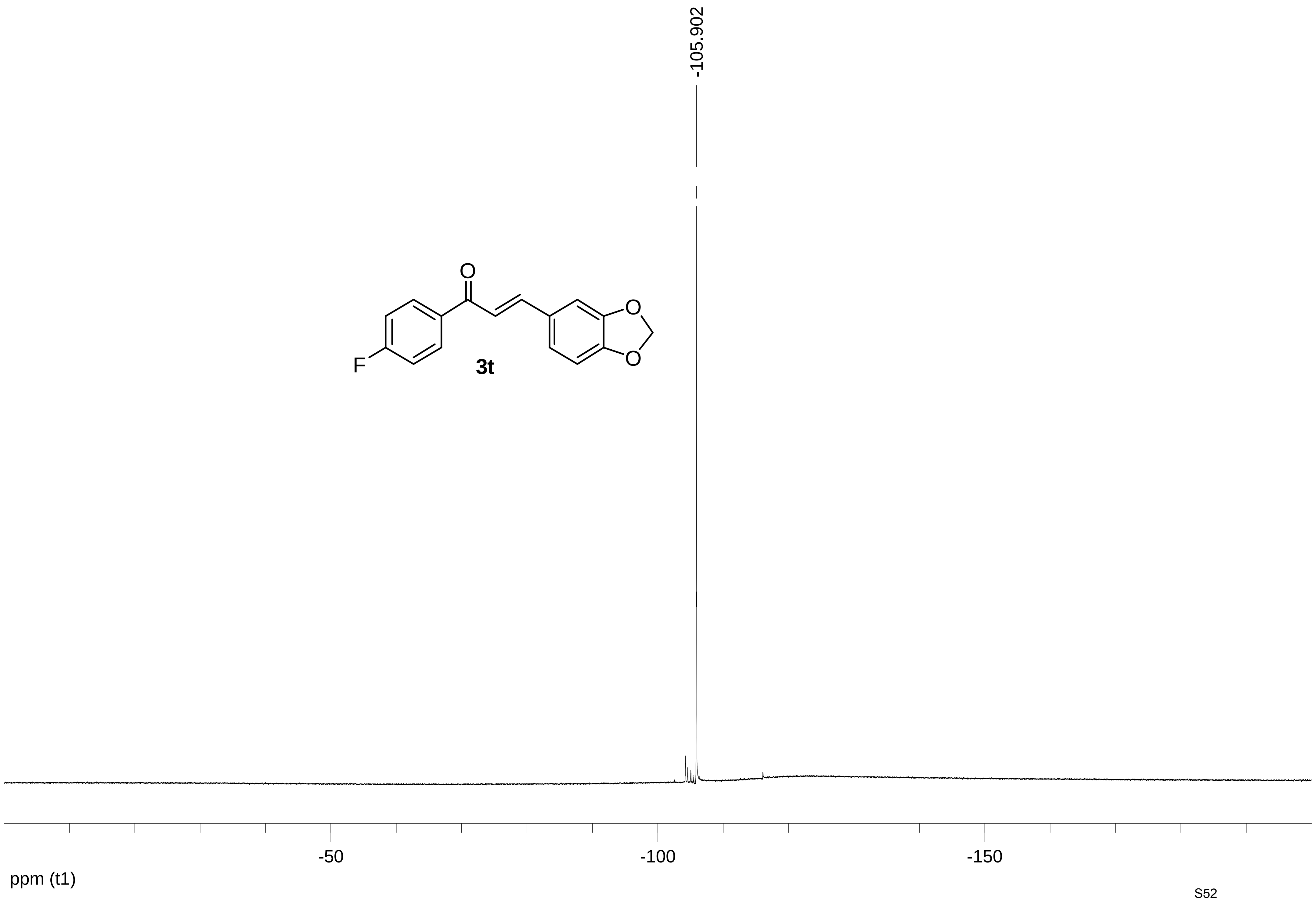
-105.902
O
F
3t
O
O
-50
-100
-150
ppm (t1)

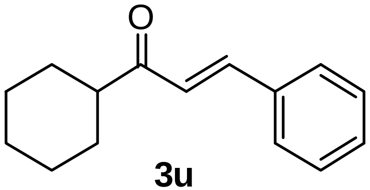

3u

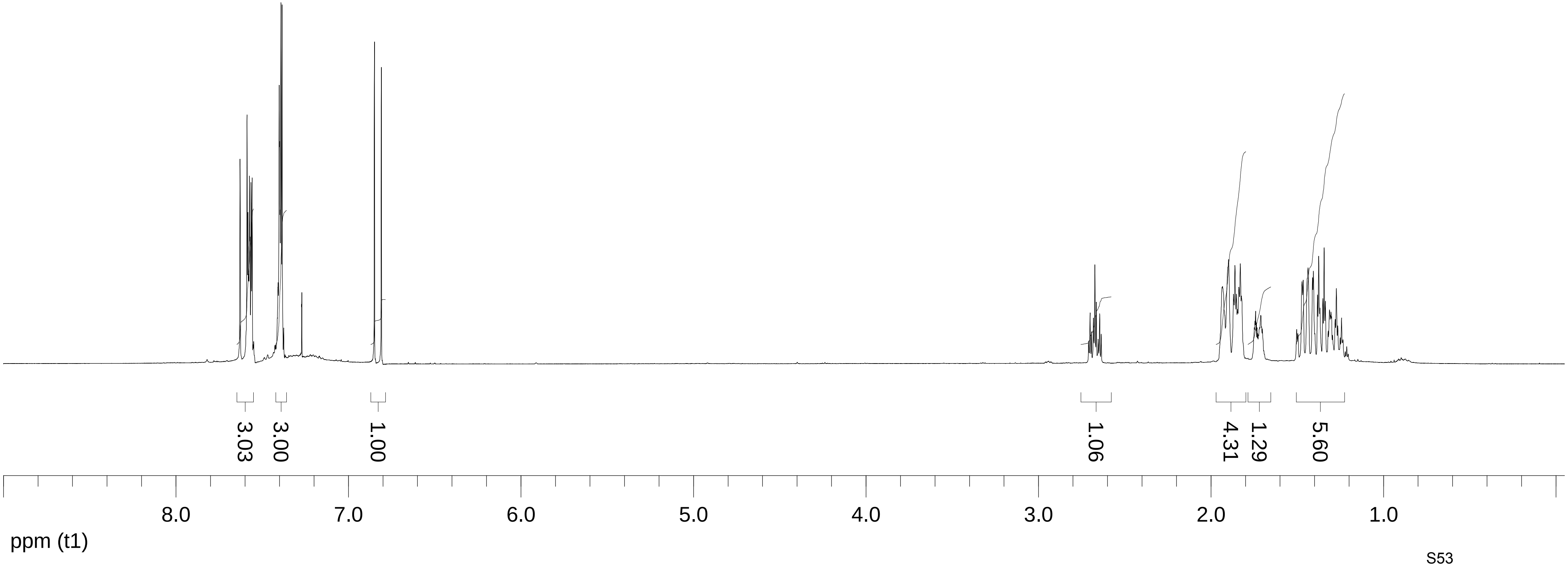

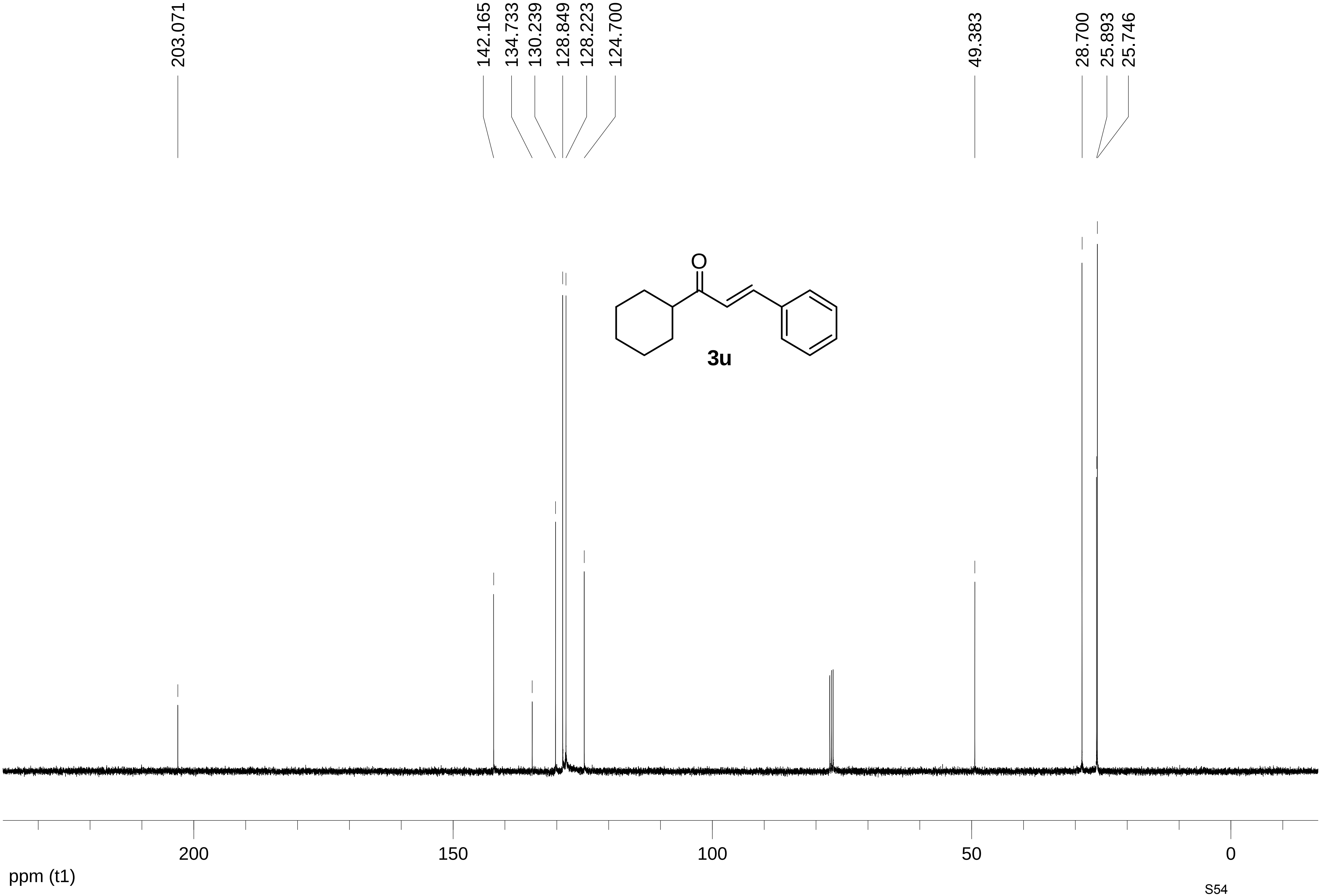
203.071
142.165
134.733
130.239
128.849
128.223
124.700
49.383
28.700
25.893
25.746
O
3u
200
150
100
50
0
ppm (t1)
